# Molecular states underlying neuronal cell type development and plasticity in the postnatal whisker cortex

**DOI:** 10.1371/journal.pbio.3003176

**Published:** 2025-05-14

**Authors:** Salwan Butrus, Hannah R. Monday, Christopher J. Yoo, Daniel E. Feldman, Karthik Shekhar

**Affiliations:** 1 Department of Chemical and Biomolecular Engineering, University of California, Berkeley, Berkeley, California, United States of America; 2 Department of Neuroscience, University of California, Berkeley, Berkeley, California, United States of America; 3 Helen Wills Neuroscience Institute, University of California, Berkeley, Berkeley, California, United States of America; 4 Center for Computational Biology, Vision Sciences and Optometry, University of California, Berkeley, Berkeley, California, United States of America; Institute for Basic Science, REPUBLIC OF KOREA

## Abstract

Mouse whisker somatosensory cortex (wS1) is a major model system to study the experience-dependent plasticity of cortical neuron physiology, morphology, and sensory coding. However, the role of sensory experience in regulating neuronal cell type development and gene expression in wS1 remains poorly understood. We assembled a transcriptomic atlas of wS1 during postnatal development comprising 45 molecularly distinct neuronal types that can be grouped into eight excitatory and four inhibitory neuron subclasses. Between postnatal day (P) 12, the onset of active whisking, and P22, when classical critical periods close, ~ 250 genes were regulated in a neuronal subclass-specific fashion when whisker experience was normal. At the resolution of neuronal types, only the composition of layer (L) 2/3 glutamatergic neurons, but not other neuronal types, changed substantially between P12 and P22. These postnatal compositional changes in L2/3 neuronal types resemble those observed previously in the primary visual cortex (V1), and the temporal gene expression changes were also highly conserved between the regions. Unlike V1, however, cell type maturation in wS1 is not substantially dependent on sensory experience, as 10-day full-face whisker deprivation from P12 to P22 did not influence the transcriptomic identity nor composition of L2/3 neuronal types. A one-day competitive whisker deprivation protocol from P21 to P22 also did not affect cell type identity but induced moderate changes in plasticity-related gene expression. Thus, developmental maturation of cell types is similar in V1 and wS1, but sensory deprivation minimally affects cell type development in wS1.

## Abstract 2

This study provides a transcriptomic atlas of the developing mouse barrel cortex, identifying temporal gene expression changes during postnatal maturation. Sensory experience minimally affects neuronal cell type maturation and conserved molecular mechanisms underlie cortical development and plasticity.

## Introduction

Neural circuits and function in the neocortex develop in a two-step sequence. Intrinsic genetic programs specify diverse cell types and organize them into an embryonic connectivity map. Subsequently, sensory experience refines this circuitry through activity-dependent mechanisms [1], including modulation of individual neuronal features such as morphology and synaptic connectivity and by altering network properties like sensory coding and population activity dynamics [2–4]. Experience-dependent plasticity exerts its largest effects during early postnatal critical periods (CPs) [5,6]. Sensory experience during critical periods likely influences the development of some neural cell populations, but how these effects differ among the hundreds of cell types that populate the mammalian cortex remains poorly explored [7–9].

Recent work in mouse primary visual cortex (V1) suggested that the maturation of glutamatergic neuronal types within the upper cortical layers (L2/3/4), but not lower-layer glutamatergic neurons or inhibitory neuronal types, is vision-dependent [10,11]. In response to visual deprivation, the transcriptomic profiles, spatial gene expression patterns, and functional tuning of L2/3 glutamatergic neurons were altered. Whether such selective influence of experience on cell type maturation is generally conserved across neocortical areas has not been studied. Given well-described deficits in experience-dependent forms of plasticity in neurodevelopmental disorders like autism, understanding the influence of experience in normal brain development is particularly important.

**Fig 1:**
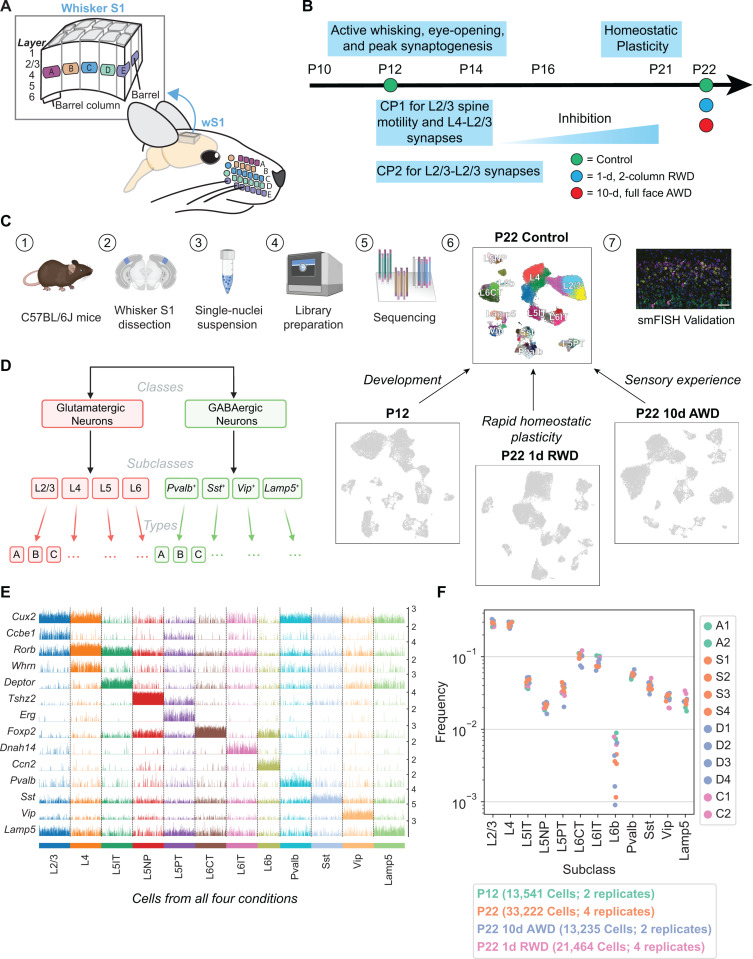
snRNA-seq atlas of the juvenile mouse primary whisker somatosensory cortex (wS1). **(A)** Schematic of the mouse whisker somatosensory system, including the facial whisker pad and the whisker somatosensory cortex (wS1). wS1 contains a somatotopic map of the whisker pad in which individual whiskers are represented by neural activity within barrel columns of the cortex. **(B)** Experimental design and developmental timeline for snRNA-seq profiling of one reference (control) dataset at P22 and three experimental conditions: an earlier time during development (P12) and following two different whisker deprivation paradigms at P22. RWD, row-whisker deprivation. AWD, all-whisker deprivation. **(C)** General experimental and computational workflow for snRNA-seq profiling and subsequent confirmatory studies. Created in BioRender. Shekhar, K. (2025) https://BioRender.com/6wszljc. **(D)** Representation of cortical neuron diversity explored in this study highlighting the three taxonomic levels: classes, subclasses, and types. Created in BioRender. Shekhar, K. (2025) https://BioRender.com/kxs5b3n. **(E)** Tracksplot showing marker genes (rows) for each neuronal subclass (columns). Data was aggregated from 81,462 nuclei across all four conditions and each subclass was subsampled to the size of the smallest subclass for plotting purposes. **(F)** Relative frequencies of neuronal subclasses are highly consistent across biological replicates and experimental conditions. The highest variance is seen for L6b glutamatergic neurons, whose frequency ranges from 0.1% to 1% of all neurons. Each biological replicate contains cells derived from three mice (see Materials and methods, S1 Data).

We studied the transcriptomic maturation of cell types in the mouse whisker primary somatosensory cortex (wS1; Fig 1A) during normal postnatal development and during deprived whisker sensory experience. wS1 processes tactile (touch) information from the facial whiskers. wS1 contains a somatotopic map of the whisker pad, so that sensory manipulation (plucking or activation) of specific whiskers drives plasticity in the cortical columns corresponding to those whiskers. This somatotopic organization provides strong technical advantages for investigating mechanisms of plasticity and has made wS1 a workhorse for studying morphological and physiological experience-dependent plasticity [3,12,13].

Cortical circuit development and critical periods are also well described in wS1 (Fig 1B). Thalamocortical axons arrive in L4 at postnatal day (P) 1–2, segregate into whisker-specific clusters at P3, and drive patterning of postsynaptic L4 neurons into modules (termed barrels). Barrel development is partly driven by neural activity during this early period (P0–3). After this age, the anatomical barrel pattern remains stable, but alterations in whisker use drive physiological plasticity in wS1 that is maximal during two overlapping critical periods (CP1: P12–P14 and CP2: P12–P16). P12 marks the onset of active whisking and coincides with peak synaptogenesis in wS1. From P12 to P14 there is a critical period during which whisker deprivation (WD) disrupts receptive field structure [14,15] and spine motility [16] in L2/3 pyramidal (PYR) cells (CP1). From P12 to P16, removing all but one whisker strengthens L4-L2/3 and L2/3-L2/3 synapses to enhance the representation of the spared whisker (CP2) [17]. Although classical critical periods close by postnatal week 3, brief 1-day WD between P21 and P22 drives homeostatic plasticity to preserve whisker-evoked firing rates in PYR cells [18]. It is unknown whether these plastic physiological changes are associated with changes in gene expression.

To study how whisker experience influences the establishment of wS1 cell types during early postnatal development, we combined single-nucleus RNA-sequencing (snRNA-seq), computational analyses, sensory perturbations, and fluorescence in situ hybridization (FISH) (Fig 1C). We first generated transcriptomic atlases of wS1 at P12 and P22, which span from the onset of active whisking through CP1 and CP2. Within each atlas, single-nucleus transcriptomes were classified into a hierarchy comprising neuronal classes, subclasses, and types (Fig 1D). We identified 250 genes with subclass-specific up- or down-regulated between P12 and P22, and validated selected temporally regulated genes using FISH. These changes overlapped significantly with developmental changes observed in V1 during a similar period [10], suggesting conserved postnatal maturation programs among cortical regions. At the neuronal cell type level, the composition of lower-layer glutamatergic and GABAergic neuronal types were largely stable between P12 and P22. Similar to V1, only L2/3 glutamatergic neuronal types underwent a significant compositional shift, indicative of postnatal maturation. Surprisingly, full-face WD from P12 to P22 had minimal impact on gene expression programs and the development of cell type composition, in contrast to V1, where dark-rearing induced significant changes in L2/3 [10,19]. However, a brief (1-d) WD of a subset of whiskers in adolescent (P21) mice led to whisker column-specific changes in the expression of immediate-early genes (IEGs) in L2/3, demonstrating plasticity-related gene regulation. Altogether, our results show that wS1 and V1 develop similar cell types with a similar developmental timeline and exhibit activity-dependent gene expression changes, but with very different effects of postnatal sensory experience on cell type development.

## Results

### A single-nucleus transcriptomic atlas of the developing mouse whisker cortex

To begin, we used droplet-based snRNA-seq to establish a reference atlas of wS1 neurons at P22 in mice with normal whisker experience. By P22, all established CPs are complete (Fig 1B, 1C). Next, to evaluate the influence of natural development and whisker experience on neuronal transcriptomic profiles, we used the same approach to profile wS1 in three additional experiments to compare with the reference atlas (Fig 1B). First, to identify temporally regulated genes and study cell type maturation from the onset of active whisking, we profiled wS1 from whisker-intact mice at P12, which coincides with peak synaptogenesis and the onset of CP1. Second, to study the role of whisker experience in guiding cell type maturation, we performed all-whisker deprivation (AWD) by plucking all whiskers bilaterally from P12 to P22, and tested how this alters the composition of cell types and/or their gene expression programs. Third, we tested whether brief (1-day) deprivation of the B and D rows of whiskers at P21 (row-based deprivation, RWD), a manipulation known to drive plasticity of excitatory and inhibitory circuits within deprived whisker columns [18,20], caused cell type-specific transcriptomic changes that could explain this physiological plasticity.

Data from each of these four experiments consisted of 2–4 snRNA-seq biological replicates, with each replicate consisting of pooled cells collected from three mice (Materials and methods). The resulting gene expression matrices were filtered to remove low-quality cells and cell-doublets [21], cells from non-neuronal populations, cells with a high proportion of mitochondrial transcripts (>1%), and cells that mapped poorly to other cortical datasets (Materials and methods). In total, we obtained 81,462 high-quality nuclear transcriptomes corresponding to neurons across the four conditions profiled (Figs 1C and S1A–S1C). We used standard dimensionality reduction and clustering approaches to hierarchically taxonomize wS1 neurons into 2 classes, 12 subclasses, and 53 molecularly distinct neuronal types. Annotations were performed by leveraging known markers, natural clustering, and supervised mapping to established cortical datasets (Fig 1D, 1E, Materials and methods). The relative frequencies of the neuronal subclasses, which spanned two orders of magnitude, were highly consistent across biological replicates within an experimental condition and across experimental conditions (Fig 1F).

### Developmental gene expression changes are subclass-specific

**Fig 2:**
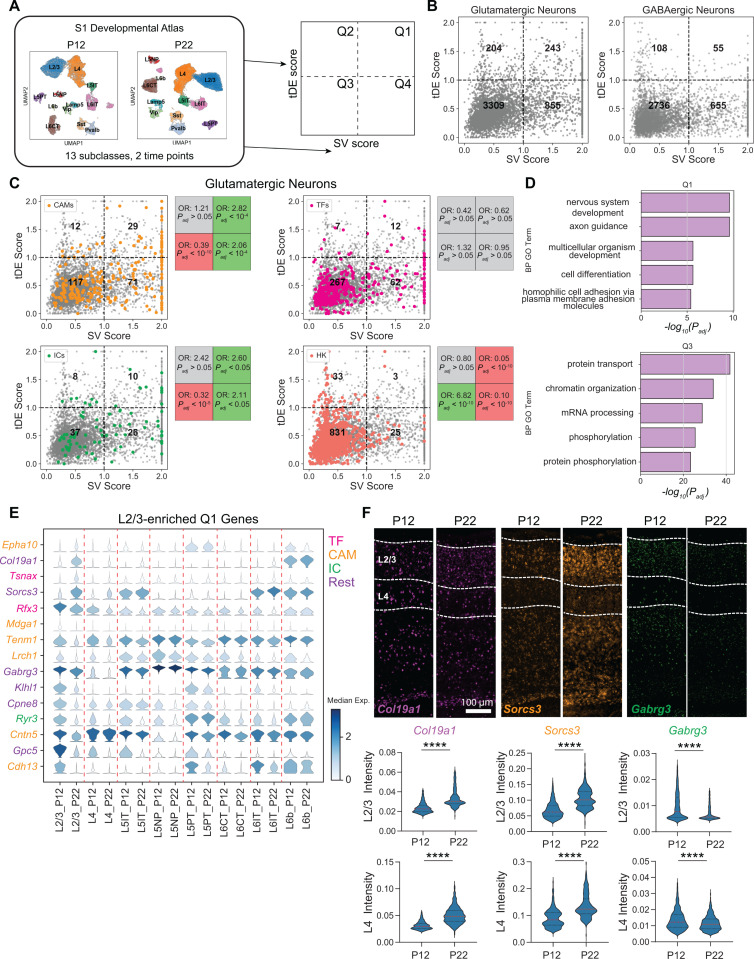
Gene expression changes between P12 and P22 are enriched in neurodevelopmental processes. **(A)** Overview of analyses to classify genes based on subclass variability and temporal differential expression. P12 contains two biological replicates, and P22 contains four biological replicates. Each replicate contains cells from three mice. **(B)** Scatter plot of subclass variability (SV) scores and temporal differential expression (tDE) scores of genes in glutamatergic (*left*) and GABAergic neurons (*right*). Scores along each axis are capped at the value of 2 (S1 Data). **(C)** Same as B for glutamatergic neurons with four gene categories highlighted. Subpanels (clockwise, starting from top left) correspond to cell adhesion molecules (CAMs), transcription factors (TFs), housekeeping genes (HKs), and ion channels (ICs). Boxes on the right of each panel list the odds ratio (OR) and adjusted p-value (*P*_adj_) for the enrichment of the corresponding gene category in each quadrant (Fisher’s exact test). Gray values indicate neither enrichment nor depletion, while red and green indicate depletion and enrichment, respectively (see Materials and methods, S1 Data for details). **(D)** Q1 is enriched in gene ontology (GO) programs associated with neurodevelopment, while Q3 is enriched in general housekeeping processes (S1 Data). **(E)** Stacked violin plot of example genes (rows) from Q1 with the highest tDE score in L2/3. Columns correspond to subclasses at P12 and P22, violins represent the expression distribution, and color represents median expression. Genes are colored according to the functional categories as in panel C (S1 Data). **(F)** FISH for tDE genes. Representative images (*top*) from an ‘across-row’ (see Materials and methods) section in wS1. Cortical layers 2/3 and 4 are indicated on the sections. Quantification (*bottom*) of mean intensity in nuclei revealed strong temporal regulation of gene expression in L2/3 consistent with what was measured with snRNA-seq (see Fig 2E). Mann–Whitney test, ****p < 0.0001. L2/3_*Col19a1*: *n* = 1,390, 1,042 nuclei, L4_*Col19a1*: *n* = 1,028, 813 nuclei, L2/3_*Sorcs3*: *n* = 1,455, 1,102, L4_*Sorcs3*: *n* = 1,021, 813 L2/3_*Gabrg3*: *n* = 1,433, 1,311, L4_*Gabrg3*: *n* = 1,026, 812. Three mice per condition, two slices per mouse (S1 Data).

To characterize gene expression patterns during normal development, we compared data from the P12 and P22 (normal experience) experiments within each cortical subclass. We computed two scores for each gene: a subclass variability (SV) score, based on its maximum expression fold change among the subclasses, and a temporal differential expression (tDE) score, based on its maximum fold change between P12 and P22 among the subclasses. A total of ~4000 genes, each expressed in >40% of cells in at least one time point and at least one subclass, were included in this analysis (S1 Table). Based on their scores (fold-change (FC) >2 along each axis), genes were stratified into four quadrants Q1–Q4 (Fig 2A, Materials and methods). Glutamatergic and GABAergic subclasses showed a similar proportion of genes (~70%–80%) with low SV and tDE scores (Q3 in Fig 2B). However, glutamatergic subclasses were > 3× more enriched than GABAergic subclasses (5.3% versus 1.5%) in genes with high subclass-variability and high tDE (Q1) and 1.5× more enriched (4.5% versus 3%) in genes with low subclass-variability and high tDE (Q2). These results indicate that glutamatergic neurons undergo greater transcriptional changes during this period of postnatal maturation.

To understand the functional significance of Q1–Q4 for glutamatergic neurons, we computed the enrichment of curated lists of transcription factors (TFs), cell adhesion molecules (CAMs), ion channels (ICs), and housekeeping genes (HKs) within glutamatergic subclasses using Fisher’s exact test (Fig 2C, Materials and methods). HKs were primarily enriched in Q3, consistent with their constitutive, non-specific expression. TFs were not significantly enriched in any quadrant, reflecting their broad role in transcriptional regulation across all subclasses during development [22,23]. CAMs, many of which regulate circuit formation [24], were strongly enriched in Q1 and, to a lesser degree in Q4, consistent with their cell type-specific and dynamic expression patterns during circuit formation reported previously in other contexts [23,25–27]. ICs were also enriched in Q1 and Q4, which aligns with physiological changes in neurons during this developmental period. Gene ontology (GO) analysis further supports these trends: Q1 genes were enriched for “cell adhesion” and “axon guidance”, while Q3 genes were associated with general cellular processes such as “protein/mRNA transport” and “chromatin reorganization” (Figs 2D and S1D). Q2 was enriched in “synaptic translation” terms, with ~60% of its enriched GO terms (8/13) overlapping with Q3 (S1D and S2A Figs). Q4 shared terms with Q1 and Q3, including “ion transport”, “cell–cell adhesion”, and “protein phosphorylation” (S2A and S1D Figs). Together, these results suggest that Q1 is a subclass-specific program related to circuit development, Q2 is a global developmental program, Q3 is a static “housekeeping” program, and Q4 is a subclass-specific program associated with neuronal identity. Similar trends were observed in GABAergic subclasses, albeit with fewer Q1 and Q2 genes (Figs 2B and S2B).

Next, we examined subclass-specific temporal gene regulation by identifying genes significantly up- or down-regulated between P12 and P22 (FC > 2 between P12 and P22, FDR < 0.05, Wilcoxon rank-sum test) (S2 Table). Of ~421 genes identified, > 60% exhibited subclass-specific expression changes (S2C, S2D Fig), with most subclass-specific genes enriched in Q1, and shared genes enriched in Q2 (Figs 2B and S2E, S2F). We detected ~ 1.5× more downregulated than upregulated genes (S2D Fig), a pattern observed in several studies of fly brain development, where gene repression plays a prominent developmental role [28–30].

Fig 2E shows examples of temporally regulated genes enriched in L2/3 glutamatergic neurons, including CAMs, TFs, ICs, and other genes. To validate the snRNA-seq findings, we performed multiplexed FISH using RNAscope (Fig 2F). We verified assay reliability using control probes. As expected, positive control probes showed widespread expression in the brain, while negative control probes produced no detectable signal (S2G, S2H Fig). We then probed three temporally regulated genes enriched in L2/3 (*Col19a1*, *Sorcs3*, and *Gabrg3*) at P12 and P22 in wS1 (Fig 2E, 2F). The pattern of temporal regulation of gene expression was consistent between snRNA-seq and FISH (Fig 2E, 2F), confirming subclass-specific temporal gene regulation in wS1 during postnatal development.

### Developmental changes in L2/3 cell type composition

**Fig 3:**
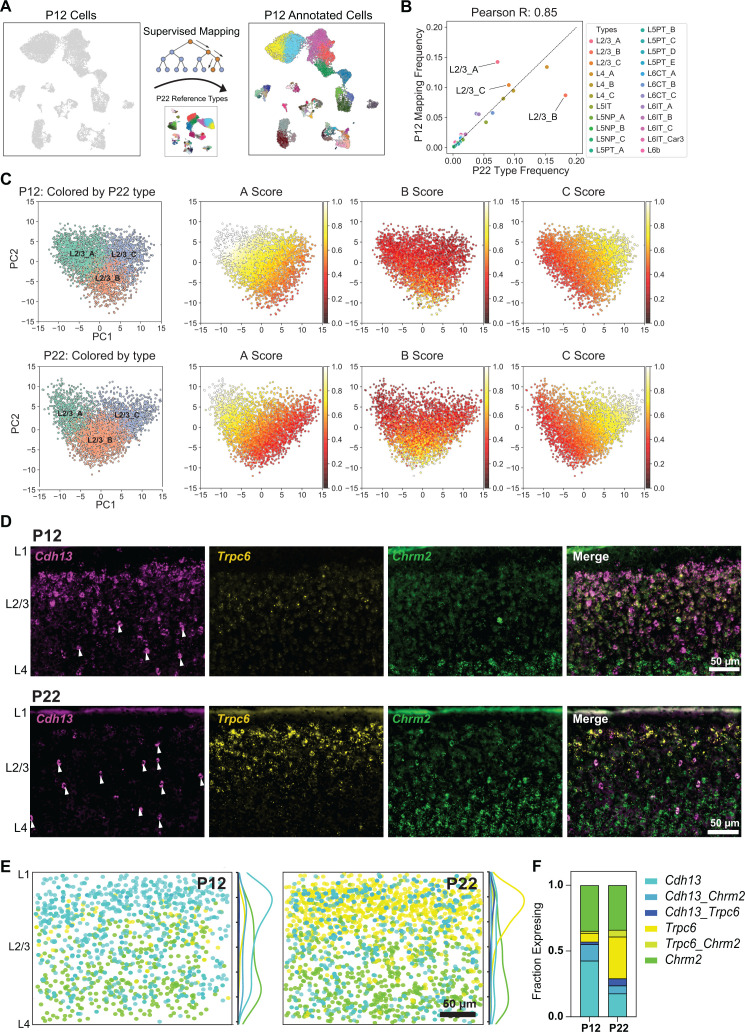
L2/3 pyramidal cell type composition and gene expression are selectively regulated during development. **(A)** Schematic for transferring P22 cell type labels (reference data) to P12 cells to facilitate cell type-level comparisons. **(B)** Within glutamatergic neurons (~80% of all neurons), all cell types except for L2/3_A and L2/3_B have approximately the same relative frequency between P12 and P22. Pearson correlation coefficient between the relative frequencies is indicated on top (S1 Data). **(C)** Scatter plot of PC1 vs. PC2 for L2/3 neurons at P12 (top row) and P22 (bottom row). Within each row, the leftmost panel highlights cells colored by their type-identity (L2/3_A, L2/3_B, and L2/3_C). In the remaining three panels within each row, cells are colored based on their aggregate expression levels of markers for each type (see Materials and methods). Between P12 and P22, L2/3_A markers decrease in expression and increase in specificity, while L2/3_B markers increase in expression during development, driving cell type identity maturation (S1 Data). **(D)** Representative FISH images showing labeling of cell type markers *Cdh13, Trpc6,* and *Chrm2* at P12 and P22 within wS1 L2/3. Arrowheads indicate putative *Cdh13*-expressing interneurons (See S5A Fig). **(E)** Summary plots based on overlay of all images of L2/3 visualizing expression of *Cdh13, Trpc6,* and *Chrm2* at P12 and P22. Circles represent individual excitatory cells within L2/3, colored based on their expression of one or more marker genes (color code as in panel E). To the right of each summary plot is the kernel density estimate for each type along the pial-ventricular axis. *Cdh13+*  cells dominate in upper L2/3 at P12, whereas *Trpc6+* cells are more abundant at P22. *N* = 10–12 slices from 3 mice per time point. **(F)** Quantification of the fraction of excitatory (*Slc17a7+*) L2/3 cells expressing one or more markers *Cdh13*, *Trpc6*, and *Chrm2* at P12 and P22. *N* = 10–12 slices from 3 mice per time point (S1 Data).

We next analyzed the maturation of cell types between P12 and P22. Using a decision tree-based classifier [31] trained on the cell type transcriptional profiles at P22, we assigned P22 type labels to each P12 transcriptome (Figs 3A and S3A, S3B; Materials and methods, S3 Table). Based on transcriptomic similarity, each neuron at P12 could be unequivocally classified into one of the 45 cell types at P22. This mapping allowed us to compare each cell type between the two ages.

Despite spanning two orders of magnitude, cell type frequencies correlated tightly between P12 and P22 within all four GABAergic subclasses and 7/8 glutamatergic subclasses (Figs 3B and S3C). This suggests that most wS1 cell types acquire a distinct and stable transcriptional signature prior to P12. The only exception was the L2/3 glutamatergic neuronal subclass (Fig 3B). Similar to what was observed in V1 [10], L2/3 glutamatergic neurons in wS1 can be clustered into three nominal types that we label L2/3_A, L2/3_B, and L2/3_C. The relative frequency of L2/3_C was similar between the two ages, but L2/3_A halved from P12 to P22, while L2/3_B doubled from P12 to P22 (Fig 3B). Despite this prominent change in cell type composition, a principal component analysis (PCA) performed using cell type markers in L2/3 glutamatergic neurons revealed that the underlying transcriptomic programs remained highly similar between P12 and P22. The top two principal eigenvectors, which accounted for ~35%–40% of the overall variance showed a 1:1 correspondence between P12 and P22 (S3D, S3E Fig), but with a slight reduction in the correlation coefficient, likely due to the compositional shift of L2/3 neurons from P12 to P22. This temporal variation in cell type frequency coincided with (i) a marked increase in the specificity of type A and C markers and (ii) increased expression strength of B markers from P12 to P22 (Figs 3C and S4A). Consistent with the shift in cell type composition, > 20% of L2/3 type-specific markers were temporally regulated, the highest among all subclasses (S4B Fig).

### Genes distinguishing L2/3 cell types and their temporal regulation

To validate the cell type compositional shift, we performed FISH in wS1, targeting the marker *Cdh13* for L2/3_A, *Trpc6* for L2/3_B, and *Chrm2* for L2/3_C (S4C, S4D Fig). These marker genes were also previously identified in V1 [10]. FISH confirmed that between P12 and P22, *Cdh13+*  L2/3_A cells decreased in frequency, *Trpc6+* L2/3_B cells increased in frequency, and *Chrm2+* L2/3_C cells remained stable (Fig 3D–3F). At P12, L2/3_A cells were concentrated at the L1/2 border, with a small population of L2/3_B cells and a large population of L2/3_C cells. At P22, L2/3_A cells were largely absent at the L1/2 border and instead concentrated in the middle of L2/3 (Fig 3E). As in V1, *Cdh13+*  cells in the center of L2/3 likely represent inhibitory neurons, as they do not co-express *Slc17a7* (vGlut1) (S5A Fig). Between P12 to P22, the proportion of *Chrm2+* L2/3_C cells remained stable, while *Trpc6+* L2/3_B cells became more prevalent compared to *Cdh13+* L2/3_A cells (Fig 3F). In contrast to V1, where L2/3_A cells remained localized to L1/2 at ~P21 despite decreasing in frequency, in wS1, L2/3_A cells decrease in frequency at the L1/2 border while *Trpc6+* L2/3_B cells become more prevalent (Figs 3F and S5B, S5C). This mirrored the compositional trends observed in the snRNA-seq data (Fig 3B). As another validation of L2/3 compositional changes, we targeted *Adamts2* for L2/3_A, *Bdnf* for L2/3_B, and *Chrm2* for L2/3C [10,32]. The expression of *Adamts2* and *Bdnf* showed the expected trend between P12 and P22 (S5D, S5E Fig), with *Adamts2+* cells decreasing in abundance while *Bdnf+* cells increased, agreeing with the snRNA-seq results (S4C Fig).

Additionally, S6 Fig highlights temporally regulated TFs, ICs, and CAMs differentially expressed among L2/3_A-C types. Although gene enrichment for each type is not strictly discrete, reflecting continuous transcriptional variation [10,19,33], some genes (e.g., *Meis2*, *Foxp1, Kcnh7, Dscaml1*) retain their expression patterns from P12 to P22, suggesting roles in the initial establishment and/or maintenance of cell type identity. Other genes (e.g., *Rfx3*, *Zbtb16*, *Scn9a*, *Ncam2*) are significantly temporally regulated and may be involved in the refinement of these cell type identities, including the maturation of their circuitry and physiology.

Taken together, our results suggest that for most wS1 neuronal types, transcriptomic distinctions and relative composition are established before P12 and persist despite developmental gene expression changes during this period of early sensory experience (Fig 2). The exceptions to this rule were L2/3 types whose composition and type-specific gene signatures are significantly altered between P12 and P22, mirroring trends reported earlier in V1 [10]. This raises the question of whether V1 and wS1 share global and subclass-specific gene expression programs related to maturation, which we now address.

### Differences and similarities between wS1 and V1 in cell type development and gene expression changes

The results thus far have highlighted multiple similarities between wS1 and V1 maturation. For example, both regions share the same transcriptomic subclasses and markers, L2/3 is selectively regulated by development in both, and both contain equivalent L2/3 cell types and markers [10]. These similarities motivated a systematic comparison of developmental gene expression changes between wS1 and V1.

**Fig 4:**
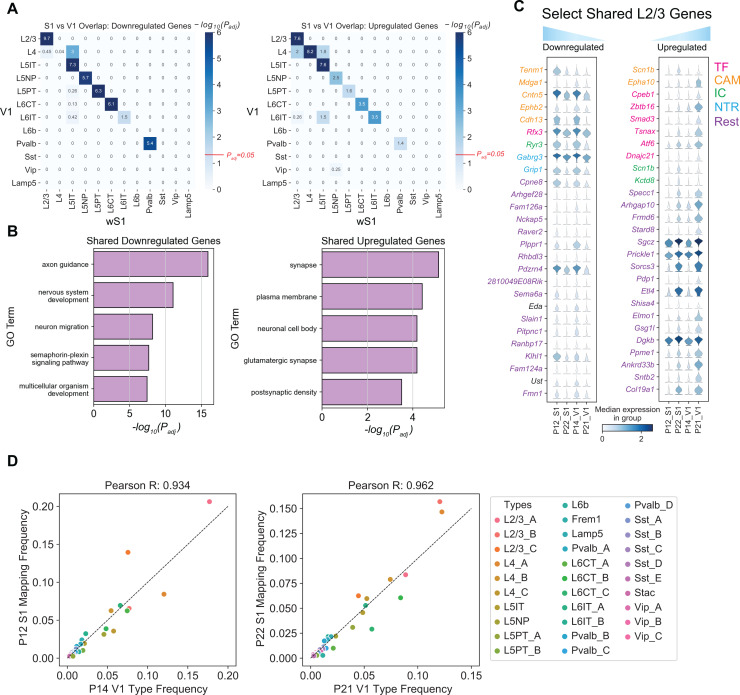
Comparative transcriptomic analysis of wS1 and V1. **(A)** Heatmap highlighting the overlap of tDE genes between all pairs of wS1 subclasses (P12 vs. P22; rows) and V1 subclasses (P14 vs. P21; columns). The left and right panels correspond to downregulated and upregulated genes. Values correspond to Bonferroni-corrected -log_10_(*P*_adj_) values from a hypergeometric test of overlap of tDE genes. The background set for these tests was the set of all the genes regulated in any subclass. Except for L4, subclasses with fewer than 10 tDE genes (S2C and S7A Figs) showed little to no overlap. The value *P*_adj_ = 0.05 is highlighted on the scalebar (right). **(B)** Top 5 GO terms from shared downregulated and upregulated genes in V1 and wS1 in corresponding subclasses from panel A. See S7C Fig for a full list (S1 Data). **(C)** Examples of shared genes that are temporally downregulated (*left*) and upregulated (*right*) in L2/3 neurons between V1 and wS1 (S1 Data). **(D)** Scatter plots showing highly similar relative frequencies between matched cell types (see S7D Fig) across V1 and wS1 (S1 Data).

We identified tDE genes in V1 between P14 and P21—the time points in the published data [10] closest to those used in this study (S4 Table). As in wS1, temporal gene regulation in V1 was predominantly subclass-specific (S7A, S7B Fig). Hypergeometric tests revealed that subclass-specific tDE genes were shared between wS1 and V1 for most subclasses (Fig 4A). For a few subclasses (e.g., inhibitory Sst, Vip and Lamp5, and excitatory L4 and L6b), there were too few tDE genes to conduct the analysis (S2C and S7A Figs). The shared subset of temporally regulated genes was enriched in GO terms related to neuronal development, including “GABAergic synapse”, “glutamatergic synapse”, “axon”, “nervous system development”, “axon guidance”, and other related programs (Figs 4B and S7C). Within L2/3, the overlapping genes were drawn from various functional categories, including CAMs, TFs, ICs, and NTRs (Fig 4C).

Moreover, wS1 and V1 share a striking correspondence in cell type composition. We trained classifiers on the V1 cell types at P14 and P21 and used them to transfer labels onto wS1 at P12 and P22, respectively. All 45 wS1 cell types mapped to the correct V1 subclass, and most cell types mapped 1:1 (S7D Fig). We also found that the relative cell type frequencies were highly concordant between the matched ages, indicating a high degree of overlap in cell type identity between the two cortical regions (Fig 4D). Together, this suggests that the two cortical regions share developmental programs at cell type resolution.

### All-whisker deprivation from P12 to P22 does not alter cell type development

**Fig 5:**
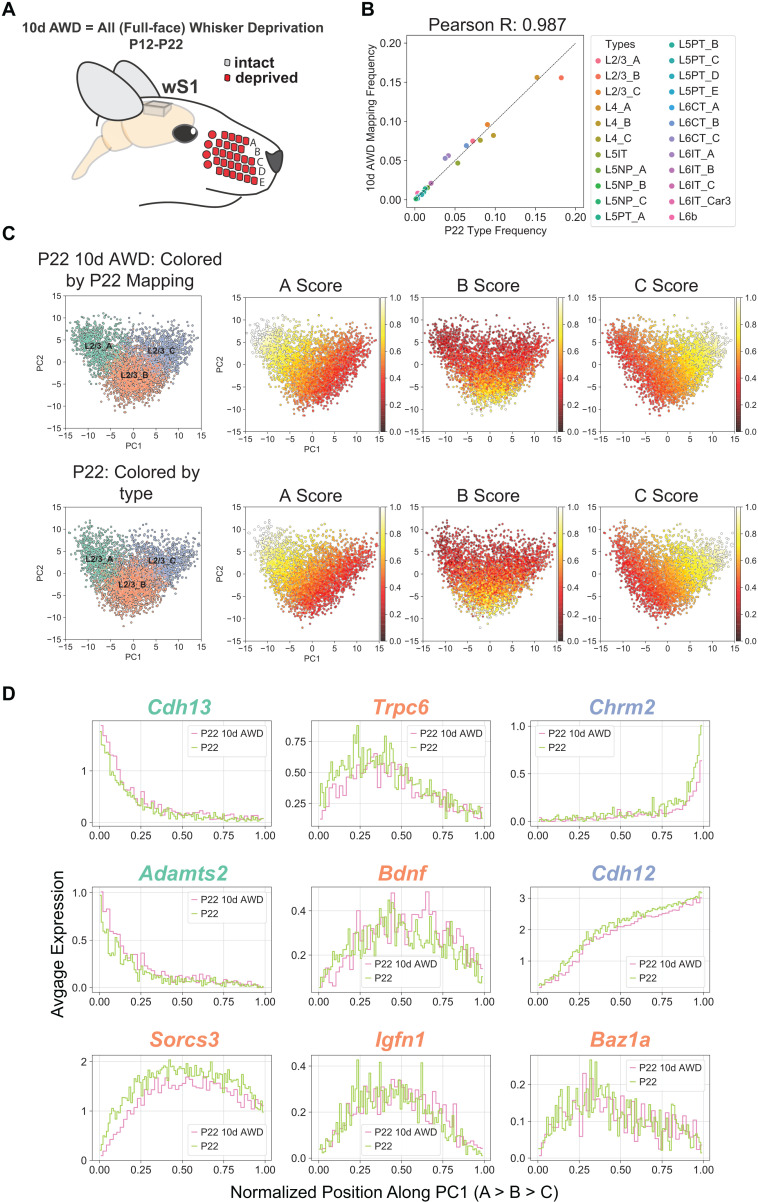
Full-face 10-day all-whisker deprivation (10d AWD) does not influence cell type maturation. **(A)** All whisker deprivation is conducted for 10 days, from P12 to P22 (10-d AWD). All whiskers on both sides of the face are plucked, then checked every other day and plucked if there is regrowth. Controls are sham mice, which are anesthetized the same amount of time as 10d AWD but not plucked. **(B)** Glutamatergic neuronal types have approximately the same relative frequency in P22 10d AWD (y-axis) and normal P22 (x-axis) (S1 Data). **(C)** PC1 vs. PC2 scatter plot for L2/3 neurons at P22 10d AWD (top) and normal P22 (bottom), highlighting the location of types and the type-specific marker scores. Representation as in Fig 3C. Scores are similar between the two datasets (see also S8D Fig) (S1 Data). **(D)** L2/3 markers genes, as in S4C Fig, are shown as a function of cells’ position along PC1 comparing patterns between P22 with normal experience and P22 10d AWD (S1 Data).

In mouse V1, 1–2 weeks of dark rearing during development selectively alters the transcriptomes of L2/3 cell types, revealing a requirement of visual experience for cell type development [10,19]. To test whether WD has a similar effect on cell type development in wS1, we plucked all whiskers on the face from P12 to P22 and analyzed the resulting gene expression changes. We refer to this manipulation as 10-day AWD (10d AWD) (Fig 5A).

We repeated the analysis outlined in Fig 3A, 3B, training a classifier on the P22 cell types and using it to transfer P22 labels to 10d AWD transcriptomes. L2/3 composition and cell type frequency at P22 were minimally affected by 10d AWD (Figs 5B and S8A). Similar to the developmental analysis in Fig 3, we used PCA to study the influence on cell type composition in 10d AWD. For normal versus 10d AWD P22, the top two principal eigenvectors, which captured >40% of the variance, exhibited a more robust 1:1 mapping compared to the case of the normal P22 versus normal P12 (S8B, S8C, and S3E Figs), consistent with 10d AWD having a minimal effect on the overall expression levels and the correlation structure of gene expression. Furthermore, the expression patterns of L2/3 type markers along the first two principal components (PCs) were highly similar between normal and 10d AWD mice at P22 (Figs 5C, 5D, and S8D).

Analysis of differentially expressed genes (DEGs) further confirmed that 10d AWD had a minimal influence on gene expression in all subclasses (S5 Table). We found 51 DEGs, which is ~ 8x smaller than the number of temporally regulated DEGs between P12 and P22 (S8E–S8G Fig). For most of DEGs, regulation was subclass-specific, with *Lamp5 +* interneurons exhibiting the largest number of changes. Altogether, we conclude that full-face WD between P12 and P22 minimally impacts the maturation of cell types and subclass-specific transcriptional programs in wS1.

### Brief row-based whisker deprivation (P21–P22) upregulates column-specific activity-dependent gene expression programs in wS1

Previous work has shown that depriving a single row of whiskers for one day induces rapid plasticity in excitatory and inhibitory circuits in the columns corresponding to the deprived whiskers. This includes Hebbian and homeostatic synaptic plasticity and changes in the intrinsic excitability of neurons, with different mechanisms at play depending on the cell type and cortical layer [18,20,34–36]. The molecular factors mediating these layer-specific changes in response to WD are unknown.

**Fig 6:**
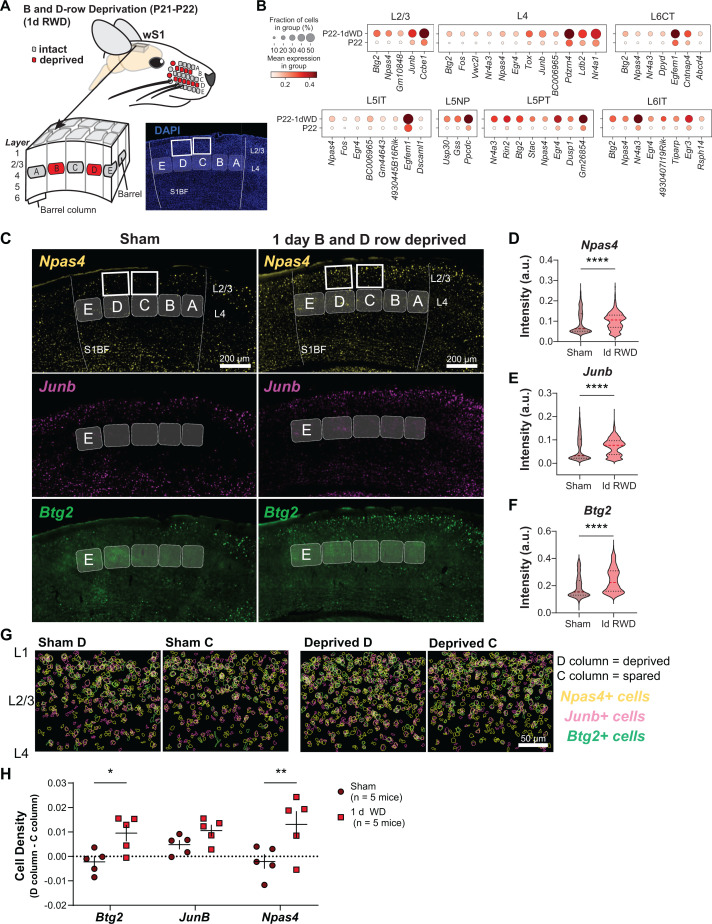
Brief row whisker deprivation (1d RWD) upregulates activity-dependent gene expression programs in deprived columns. **(A)** Schematic representation of the 1d row-whisker deprivation (RWD) manipulation and ‘across-row’ S1 section in which all barrel columns are identifiable. A representative image of DAPI labeling in ‘across-row’ S1 section. **(B)** Dotplots of genes upregulated by 1d RWD in glutamatergic subclasses (panels). Within each panel, rows indicate condition and columns indicate genes. The size of each circle corresponds to the % of cells with nonzero expression, and the color indicates average expression level (S1 Data). **(C)** Representative wide-field images of wS1. Barrels and barrel fields are indicated with light gray rectangles and labeled. Boxes indicate the locations of ROIs used for analysis in G. **(D)** *Npas4* intensity inside *Slc17a7*+  L2/3 excitatory cells is increased after 1d RWD. Violin plots show median (dashed line) and quartiles (dotted lines) across individual cells. Mann–Whitney test, *p* < 0.0001 ****, *n* = 1,852, 1,699 cells, respectively, from 5 mice per group (S1 Data). **(E)** *Junb* intensity inside *Slc17a7*+ L2/3 cells is increased after 1d RWD. Mann–Whitney test, *p* < 0.0001 ****, *n* = 1,864, 1,703 cells, respectively, from 5 mice per group (S1 Data). *(F)* *Btg2* intensity inside *Slc17a7*+ L2/3 cells is increased after 1d RWD. Mann–Whitney test, *p* < 0.0001 ****, *n* = 1,836, 1,706 cells respectively, from 5 mice per group (S1 Data). **(G)** L2/3 C and D columns were compared to examine whether gene expression changes are specific to the deprived (D) column. Pseudo-colored outlines of *Npas4, Junb, and Btg2* expressing cells. Each plot is an overlay of 5 images of L2/3 S1 from 3 mice. Only *Slc17a7+* cells are shown. **(H)** Quantification of column-specific gene expression changes in 1d RWD. Plotted values are the difference between the fraction of *Npas4/Junb/Btg2* expressing cells among all excitatory (*Slc17a7*+) cells in the L2/3 D column and the corresponding fraction in the L2/3 C column. Symbols represent individual sham or 1d RWD mice. For each condition, mean and SEM (error bars) are shown. Two-way RM-ANOVA, Whisker Experience: *F*(1,8) = 11.41, **p = 0.0097 with Šidák correction for multiple comparisons, *Npas4*: **p = 0.002. *JunB*: n.s., *p* = 0.20. *Btg2*: *p = 0.014 (S1 Data).

We performed 1-day deprivation of the B and D whisker rows on P21 (1d RWD), a manipulation that induces competitive whisker map plasticity [18], and assessed transcriptomic cell types and gene expression changes in S1 using snRNA-seq and FISH at P22 (Fig 6A). Cell type distributions were highly correlated between normal and 1d RWD datasets at P22 (S9A, S9B Fig). As in the normal and 10d AWD P22 datasets, variation within L2/3 neurons was captured well by the top two PCs (S9C Fig), which correlated 1:1 with the top two PCs of normal P22 data (S9D Fig). Furthermore, the expression patterns of L2/3 type markers along the first two PCs were highly similar between normal and 1d RWD mice at P22 (S9E–S9G Fig). Altogether, this suggests that 1d RWD has a minimal influence on cell type identity and composition in wS1, as expected for such a brief sensory manipulation.

It is well-established that sensory manipulation induces activity-dependent gene expression programs that result in long-lasting cellular adaptations essential for learning and maintaining circuit homeostasis [37,38]. We hypothesized that 1d RWD may elicit an activity-dependent transcriptional response (Fig 6A). Within each neuronal subclass, we sought genes expressed in at least 20% of cells that were upregulated in 1d RWD mice (FC > 2, FDR < 0.05, Wilcoxon rank-sum test) (S6 Table). We detected 59 such genes across all 12 subclasses. Fig 6B shows that the upregulated genes contain well-known “activity-dependent” genes such as *Npas4*, *Btg2*, *Junb*, and *Nr4a1* [37]. Importantly, these genes were different from the few regulated by 10d AWD (S8 Fig), indicating their specificity to 1d RWD plasticity.

We performed FISH experiments to confirm these gene expression changes and assess their laminar and columnar organization in wS1 (Fig 6C). We used a plane of section that contains one whisker column from each of the five rows (A–E), enabling whisker row identity to be recognized since each whisker row maps to an array of cortical columns in wS1 [39] (Fig 6A). We probed for three IEG candidates shown in Fig 6C: *Npas4*, *Btg2*, and *Junb*. These mRNAs were significantly increased in L2/3 of wS1 as measured by intensity per L2/3 excitatory cell (Fig 6D–6F). To determine if the mRNAs were upregulated in a whisker column-specific manner, we separately analyzed the number of cells expressing each mRNA in excitatory L2/3 neurons in the deprived D versus non-deprived C column of the same tissue section from sham and deprived mice (Fig 6G, 6H). *Npas4+* and *Btg2+* cells were significantly upregulated in L2/3 of the D column compared to L2/3 of the C column in whisker-deprived mice but not in sham controls. This indicates whisker column-specific gene regulation during competitive map plasticity (Fig 6H). *Junb+* cells showed a non-significant trend toward increase in D compared to C in 1d RWD mice. This suggests that the elevation in overall *Junb* intensity in L2/3 (Fig 6E) may be less column-specific than for *Npas4* and *Btg2*. Together, these results suggest that 1d RWD triggers rapid and selective induction of genes functionally associated with activity-dependent plasticity in a spatially localized pattern wherein mRNAs are more strongly upregulated in the deprived columns than in neighboring spared columns. Such gene expression patterns may constitute an early molecular mechanism of competitive map plasticity.

## Discussion

### A postnatal developmental atlas of mouse wS1

Previous studies have profiled transcriptomic cell diversity in the adult and adolescent somatosensory cortex (wS1) [7,40–42], a canonical model for studying cortical development and plasticity, but early postnatal gene expression changes shaping cell type identity remain unexplored. Here, we used snRNA-seq to analyze ~80,000 single-nucleus transcriptomes in mouse wS1 across P12 and P22 and three rearing conditions. The taxonomy and frequency distribution of ~45 neuronal cell types were conserved across time points and conditions, enabling identification of shared and cell type-specific changes during normal development and WD. Comparing P12 and P22, we identified ~250 temporally regulated genes, with over 60% being subclass-specific and linked to neuronal maturation processes like axon guidance and synapse formation. Glutamatergic neurons exhibited greater temporal regulation than GABAergic neurons, consistent with their increased evolutionary divergence [43]. By uncovering the molecular programs underlying postnatal development of sensory cortex, our research can inform understanding of neurodevelopmental disorders such as autism that are associated with circuit dysfunction and impaired sensory processing.

### L2/3 cell types undergo significant changes during normal development

Of the 45 molecularly defined neuronal cell types, 42 were present at identical frequencies at P12 and P22, indicating that most wS1 neuron types are defined before P12. The only cell types that undergo significant changes in composition and cell type-specific gene expression between P12 and P22 are PYR cell subtypes in L2/3 (L2/3_A-C). The molecular identity and developmental changes of the three L2/3 cell types in wS1 mirror those observed in V1 [10], where L2/3 cell types have been shown to possess unique binocular tuning properties and correlate with distinct projections to higher visual areas [10,44]. It is therefore possible that the three L2/3 cell types in wS1 also have distinct functional properties and connectivity patterns. For example, L2/3_A cells were enriched in *Cdh13*, while L2/3_B/C cells were enriched in *Cdh12*. Because L5 PYR cells that express *Cdh12* or *Cdh13* are inhibited by CCK + versus PV+ interneurons, respectively [45], L2/3_A cells and L2/3_B/C cells may show a similar inhibitory circuit specialization. Similarly, in adult mouse S1, L2/3 cells enriched in the *Baz1a* gene (likely corresponding to our L2/3_B type) undergo robust changes in IEG expression in response to whisker input [46]. The delayed maturation that we observed for L2/3_B cells may be related to this pronounced experience-dependent plasticity in adulthood. Finally, the segregation of L2/3_A, B, and C cell types at different laminar positions suggests that the L2-situated cells (L2/3_A and B) may receive less VPM thalamocortical axonal innervation than L3-situated cells (L2/3_C), where VPM axon density is higher [47,48], as well as different local inhibitory inputs [49].

A potential mechanism underlying the developmental flexibility of L2/3 neurons could be the presence of a transcriptomic gradient. In PC space, we observed that L2/3 neurons form a triangle, with each of the three cell types occupying a vertex. Additionally, many markers were expressed in a gradient fashion, like the functional gradients observed for L2/3 neurons in V1 [50]. This suggests that wS1 L2/3 neurons exhibit an overall gradient pattern while also containing some specialist cell types, mirroring what has been observed in V1 and the intestine [10,19,51]. Occupying a flexible continuum could facilitate the drastic gene expression and compositional shifts observed in these neurons from P12 to P22.

### Developmental transcriptomic conservation and divergence between V1 and wS1

Recent studies have shown a high degree of correspondence in neural diversity and cell type markers between cortical areas [52,53], and this was indeed the case between wS1 and V1. Our comparison of P12 to P22 in wS1 and P14 to P21 in V1 revealed similar developmental gene expression patterns. In most cases, there was significant overlap in the up- and down-regulated genes between matched neuronal subclasses between the two regions and little to no overlap across distinct subclasses. The notable exception was that L4 neurons did not share downregulated genes between the two regions (Fig 4A), which may be related to the fact that L4 contains spiny stellate cells in wS1 but not V1.

Despite this broad similarity, there was a laminar difference in the organization of L2/3 neuron subclasses between the two regions. Our data suggests that wS1 undergoes a similar remodeling of cell types from P12 to P22 as V1, with A-like cells decreasing and B-like cells increasing in number. However, unlike V1 where the L2/3_A-C cells segregate into sublaminae along the radial (pial-ventricular) axis at P21, we found that, in wS1, L2/3_A and L2/3_B somas are intermixed in superficial L2/3, but are spatially separated from the C somas, which reside in deep L2/3. The factors driving this difference between V1 and wS1 are unknown but may stem from broader differences in laminae across the sensory cortices [54].

The overall similarity in gene expression programs between V1 and wS1 is expected, as both regions share comparable timelines of postnatal development. Thalamocortical axons innervate L4, and callosal axons cross the midline and undergo activity-dependent rearrangement at similar stages in both regions [55,56]. Adult patterns of sensory input emerge in wS1 and V1 around the same time, with onset of active whisking at P12 and eye-opening at P14. However, the earliest sensory-evoked responses appear earlier in wS1, in the first few postnatal days [57], than in V1, which requires eye opening. Critical periods also differ, with classical critical periods for WD-induced plasticity in wS1 closing by ~ P16, while ocular dominance plasticity in V1 persists until P32 [13,58,59].

Despite the importance of sensory experience in wS1 circuit function, our findings reveal that WD from P12 to P22 does not impact cell type development in wS1, unlike dark rearing in V1, which alters gene expression and cell type composition in L2/3 [10,19]. This suggests that intrinsic programs may play a larger role in wS1 cell type maturation [60]. Why might L2/3 cell type development be regulated by visual experience in V1, but not by 10d AWD WD in wS1? One possibility is that the overall impact of sensory experience on cortical cell and circuit development is milder in wS1 than in V1. Consistent with this idea, ocular dominance shifts of V1 neurons driven by monocular deprivation are often stronger than whisker tuning shifts in wS1 driven by plucking a subset of whiskers [61,62]. During the critical period for ocular dominance plasticity in V1, visual experience shapes thalamocortical axons. It is required to maintain their structure, while in wS1, whisker sensory use does not alter thalamocortical or L4 topography after P4, and before that only nerve or follicle damage is sufficient to drive plasticity [56,63]. The critical period for ocular dominance plasticity has a stark ending at P32 in mice [58], while S1 retains many forms of experience-dependent plasticity into adulthood [3], suggesting that wS1 plasticity may not be as tied to developmental mechanisms as in V1.

Differences in sensory input deprivation paradigms may also explain this discrepancy [13]. Dark rearing in V1 completely eliminates visual sensory drive and statistical patterns of sensory input, leaving only retinal spontaneous activity, which has different properties. On the other hand, whisker plucking reduces but does not fully remove tactile input. For example, grooming and cuddling still provide afferent activation. Thus, plucking is likely to eliminate less sensory input than dark rearing and, hence, may have a less pronounced impact on S1 gene expression.

### Competitive whisker deprivation drives plasticity-related gene expression programs in L2/3

Despite the lack of requirement for whisker experience on cell type maturation, we found that brief WD of only two rows of whiskers (1d RWD) upregulates activity-dependent gene expression programs in multiple wS1 cell types, including *Npas4, Btg2, Junb, and Nr4a1*, which are known to be involved in synaptic plasticity and circuit homeostasis [37]. Typically, expression of these IEGs is thought to be driven by increased levels of activity, as occurs during environmental enrichment (P65-75 animals) in S1 [64,65] or dark rearing followed by light exposure in V1 [66]. Interestingly, dark-rearing alone also increased the expression of several genes, particularly in L2/3 excitatory neurons [66]. Our finding that a subset of IEGs are upregulated in response to deprivation indicates that IEG induction may represent a general plasticity program broadly used to engage plasticity mechanisms rather than a specific consequence of elevated neural activity.

We found that deprived columns showed increased expression of IEGs relative to neighboring spared columns. This deprivation paradigm triggers synaptic plasticity, a component of competitive map plasticity, wherein whisker-evoked responses to deprived whiskers weaken and shrink within the whisker map while responses to spared whiskers strengthen and expand [67]. Thus, the selective upregulation of IEGs in deprived columns may represent an early molecular step underlying competitive map plasticity. Together, the 1d RWD results indicate that whisker experience exerts only relatively subtle effects on the development of cell types but impacts gene expression levels within specific cell types.

### Summary and limitations

This work contributes a single-nucleus transcriptomic resource for postnatal wS1 development under normal and deprived sensory conditions. We delineated developmental changes in neuronal subclasses and types, and found that these were highly conserved between mouse V1 and wS1. However, whisker experience served a limited role in wS1 cell type development, which contrasts with the greater role of vision in V1 cell type development. Brief WD induced significant whisker column-specific changes in the expression of IEGs. Thus, while wS1 may rely less on experience and more on hardwired genetic programs than V1 to develop cell types, these cell types can undergo experience-dependent gene expression changes.

Throughout the study, we validated multiple scRNA-seq findings by performing FISH experiments targeting representative genes. Future studies could leverage spatial transcriptomics to quantify gene expression in wS1 barrels to further test the hypotheses established in this work. More research is needed to assess the gene regulatory networks, including chromatin organization and DNA methylation, that mediate experience-driven cell-type-specific transcriptome changes. Moreover, low sampling resolution or protein level changes could explain the limited transcriptomic effects observed during 10d AWD or 1d. Finally, integrating cell type-specific IEG expression with neuronal physiology using slice electrophysiology or in vivo two-photon imaging during whisker sensory behaviors could provide deeper insights into the functional consequences of gene expression changes.

## Materials and methods

### Ethics statement

All methods, including the handling of vertebrate animals, followed NIH guidelines and were approved by the UC Berkeley Animal Care and Use Committee under Protocol ID Number AUP-2016-02-8351-3.

### Computational methods

#### Alignment and gene expression quantification.

FASTQ files with raw reads were processed using Cell Ranger v7.0.1 (10× Genomics) with default parameters. We used the mm10 (GENCODE vM23/Ensembl 98) reference genome and transcriptome. Reads aligning to the entire gene body (exons, introns, and UTRs) are used to quantify expression levels. Each single-nucleus library was processed using the same settings to yield a gene expression matrix (GEM) of mRNA counts across genes (columns) and nuclei (rows). Each row ID was tagged with the replicate metadata for later meta-analysis. We henceforth refer to each single-nucleus transcriptome as a ‘‘cell.’’

#### Initial pre-processing and quality control of snRNA-seq data.

This section outlines the initial transcriptomic analysis of data from all replicates. Unless otherwise noted, all analyses were performed in Python using the SCANPY package [68] based on the following steps https://github.com/shekharlab/wS1dev:

1. Raw GEMs from 12 snRNA-seq libraries were combined: P12 control (A1, A2), P22 control (S1, S2, S3, S4), P22 1d RWD (D1, D2, D3, D4), and P22 10d AWD (C1,C2). This resulted in a GEM containing 199,524 cells and 32,285 genes.
2. We generated scatter plots of the number of transcript molecules in each cell (*n*_counts_), the percent of transcripts derived from mitochondrially encoded genes (percent_mito), and the number of expressed genes (*n*_genes_) to identify outlier cells. Cells that satisfied *n*_genes_ < 7,000 and *n*_counts_ < 40,000, and *n*_counts_ > 500 were retained, and genes detected in fewer than 100 cells were removed from further analysis. This resulted in a GEM of 199,074 cells and 21,258 genes (S1A Fig). Cells were normalized for library size differences by rescaling the transcript counts in each cell to a total of 10,000, followed by log transformation.
3. For clustering and visualization, we followed steps described previously [10]. Briefly, we identified highly variable genes (HVGs), *z*-scored expression values for each HVG across cells, and used the z-scored data to compute a reduced-dimensional representation based on PCA. The top 40 PCs were used to build a nearest-neighbor graph on the cells. We clustered this graph using the Leiden algorithm [69] and embedded it in 2D via the Uniform Manifold Approximation and Projection (UMAP) algorithm [70]. We identified 39 preliminary clusters.
4. Four of the 39 preliminary clusters contained 20%–40% mitochondrial transcripts, while the remaining clusters contained, on average, 5%–10% mitochondrial transcripts. We removed these clusters from the data, resulting in 169,674 cells (S1A Fig). One cluster belonged to biological replicates S1/S2, another to A1/A2, another to C1/C2/S4, and the last one to S3/D3/D4.
5. With this filtered set of 169,674 cells, we re-ran the clustering pipeline described above to obtain a new set of clusters. To assess the quality of these clusters, we trained a multi-class classifier using XGBoost, a gradient boosted decision tree-based classification algorithm [31] on the subclasses from the primary visual cortex [10] (V1). From the V1 study, we used all of the postnatal time points collected from normally reared animals. Twenty-eight out of the 39 preliminary clusters mapped strongly to one of the 20 V1 subclasses. However, 11 clusters mapped diffusely to the subclasses and/or mapped with low classifier confidence. Further inspection indicated that the poorly mapping clusters had higher doublet scores than tightly mapping clusters [21], and their top markers were enriched in GO terms related to “apoptosis” and “response to toxic substance”. Removal of the 11 problematic clusters reduced the number of cells to 114,812. Next, we examined the V1 subclass composition of each remaining cluster and removed cells corresponding to any V1 subclass that accounted for <2% of the cluster. This further purified clusters, ultimately yielding 112,233 cells (S1A Fig).
6. Finally, we isolated each of the four experimental conditions (P12 control, P22 control, P22 1d RWD, and P22 10d AWD) and re-ran the clustering pipeline described above. Within each condition, we removed clusters containing cells that mapped to V1 subclasses in a non-specific manner. This yielded a final number of 111,299 cells that form the foundation of this paper (S1A Fig). Only the 81,462 neurons were used in all the analyses reported in this paper.

#### Temporal regulation and subclass specificity analysis.

The gene quadrant analysis (Fig 2) was performed on the P12 and P22 control datasets separately for the glutamatergic and GABAergic neuronal classes. Within either class, we performed a Wilcoxon rank-sum test for each subclass against the rest of the subclasses to identify subclass-specific markers (FC>2, FDR < 0.05, expressed in >40% of cells). For each pair of gene and subclass, we calculated fold changes separately at P12 and P22 and selected the maximum value as the SV score. We next performed a Wilcoxon rank-sum test between P12 and P22 for each subclass to identify temporally regulated genes (FC > 2, FDR < 0.05, expressed in >40% of cells). The maximum temporal FC value for each gene across all subclasses was assigned to be its tDE score. To define the quadrants, a threshold FC value of 2 was chosen, and we verified that values between 1.5 and 2.5 do not qualitatively impact the results shown in Figs 2 and S2.

In Fig 2B, 2C of the main text, we assess the statistical enrichment of specific gene groups in each quadrant using a Fisher’s Exact Test. For each quadrant, we calculated the odds ratio (OR) and *p*-value using Fisher’s Exact Test to determine whether TFs, CAMs, ICs, or HKs were significantly over- or under-represented compared to the null expectation based on all genes. Given the multiple comparisons, we applied the Bonferroni correction to adjust the *p*-values, controlling for false positive rate. The results were visualized by categorizing the quadrants based on statistically significant under- or over-representation: quadrants were shaded gray if the adjusted *p*-value exceeded 0.05 (no enrichment), red if the adjusted *p*-value was less than 0.05 with OR < 1 (significant under-representation), and green if the adjusted *p*-value was less than 0.05 with OR > 1 (significant over-representation). This approach allowed us to identify statistically significant enrichment patterns, highlighting their potential regulatory roles in wS1 development. Finally, GO enrichment analysis was performed on each quadrant’s set of genes using the python package GOATOOLS [71]. We used the default background set in GOATOOLS, which comprised all protein-coding genes.

#### Subclass-by-subclass differential gene expression analysis between each experimental condition and P22 control.

We sought to identify gene expression differences at the subclass level between the P22 control dataset and the three experimental datasets, including P12, P22 10d AWD, and P22 1d RWD. Here, each subclass was isolated, and a Wilcoxon rank-sum test was performed for that subclass between P22 control and each of the three conditions. Of the genes expressed in >40% of cells in one of the two datasets, we selected those with FC > 2 at false-discovery rate (FDR) <0.05. A python implementation of the R package UpSetPlot [72] was used to compute and visualize the number of genes regulated in a shared and subclass-specific fashion. For these differential gene expression tests, biological replicates S3, D3, and D4 were excluded due to their relatively low number of transcript counts and expressed genes to not affect the results (S1B, S1C Fig). However, these replicates were not excluded from any other analysis and consistently contained all neuronal subpopulations with no frequency discrepancies (Fig 1F).

#### Class, subclass, and cell type annotation of snRNA-seq data.

We annotated cells in our dataset according to the taxonomy in Fig 1D. The two major neuronal classes were easily identified using glutamatergic marker *Slc17a7I* and GABAergic markers *Gad1* and *Gad2* in each condition separately. Additionally, we identified non-neuronal groups using known markers [32]. Non-neuronal cells were discarded at an early stage, and we focused on the two neuronal classes, which clustered distantly from each other in gene expression space. Clusters within each condition naturally separated according to subclasses that could be annotated based on established markers [10]. The class and subclass levels of the taxonomy are highly conserved across cortical regions and studies. However, as the cell type level tends to vary across regions, studies, and conditions, we annotated cell types in the P22 control dataset, and then transferred these labels onto the other conditions.

To annotate cell types in the P22 control dataset, we first trained a classifier on the cell types of the P21 V1 dataset [10]. We then applied the classifier to the P22 control dataset to assign each wS1 neuron a P21 V1 cell type label. Second, we isolated each subclass and used Leiden clustering [69], varying the resolution parameter from 0.25 to 2, with higher values providing more clusters. Third, we trained and validated a classifier for each clustering resolution, identifying the resolution at which cluster validation error (computed using held-out cells) increases significantly, indicating over-clustering. The general procedure to train and validate such classifiers is described in the following section. Another telltale sign for diagnosing over-clustering was when differential expression analysis yielded highly overlapping marker sets for their clusters. Ultimately, these steps nominated a range of optimal clustering resolutions, and we chose the final resolution at which the validation error was low (90%) and there was a high concordance with the V1 clustering. This resolution also yielded unique marker sets across the cell types.

Finally, we combined all subclasses for each of the two classes (glutamatergic and GABAergic neurons) and used a classification analysis to verify that we had not over-clustered the data. We trained a classifier on the P22 wS1 control data and mapped the P21 V1 data to it. Clusters from the P22 wS1 data were removed if they satisfied the following criteria simultaneously: (1) received no mapping from V1, (2) had a high doublet and/or mitochondrial score, and (3) were not learnable during training. This procedure filtered ~800 cells, approximately 2.5% of the P22 control data.

#### Classifier-based mapping of experimental conditions to P22 control data.

To assess transcriptomic correspondence of clusters across ages (P12 versus P22) or between rearing conditions (control versus 1d RWD and 10d AWD), we used XGBoost, a gradient boosted decision tree-based classification algorithm [31]. These analyses were performed to study the effects of development (P12), sensory experience (P22 10d WD), and rapid homeostatic plasticity (P22 1d WD) on cell type identity and composition. We also used this approach to compare the cell type compositional differences between V1 cell types and wS1 cell types. In a typical workflow, we trained an XGBoost (version 1.3.3) classifier to learn subclass or type labels within the P22 control “reference” dataset and applied this classifier to another “test” dataset. The correspondences between cluster IDs and classifier-assigned labels for the test dataset were used to map subclasses or types between datasets. The classification workflow is described in general terms below and applied to various scenarios throughout the study.

Let *R* denote the reference dataset containing *N*_*R*_ cells grouped into *r* clusters. Let *T* denote the test dataset containing *N*_*T*_ cells grouped into *t* clusters. Each cell is a normalized and log-transformed gene expression vector ***u*** ∈ *R* or ***v*** ∈ *T*. The length of ***u*** or ***v*** equals the number of genes. Based on clustering results, each cell in *R* or *T* is assigned a single cluster label, denoted cluster(***u***) or cluster(***v***). cluster(***u***) may be a type or subclass identity, depending on context.

The main steps are as follows:

1. We trained a multi-class XGBoost classifier *C*_*R*_^*0*^ on *R* using the intersection of HVGs from *R* and *T* as features. The training dataset was split into training and validation subsets. For training, we randomly sampled 70% of the cells in each cluster, up to a maximum of 1,000 cells per cluster. The remaining “held-out” cells were used for evaluating classifier performance. Clusters with fewer than 100 cells in the training set were upsampled via bootstrapping to 100 cells to improve classifier accuracy for the smaller clusters. Classifiers achieved >95% accuracy or higher on the validation set for most clusters, with some clusters yielding 85%−95% accuracy. XGBoost parameters were fixed at the following values:
  1. ‘Objective’: ‘multi:softprob’
  2. ‘eval_metric’: ‘mlogloss’
  3. ‘Num_class’: *r*
  4. ‘eta’: 0.2
  5. ‘Max_depth’: 6
  6. ‘Subsample’: 0.6
2. When applied to a test vector ***c***, the classifier *C*_*R*_^*0*^ returns a vector *p = (p*_*1*_*, p*_*2*_*, …)* of length *r*, respectively. Here, *p*_*i*_ represents the probability value of predicted cluster membership within *R*, respectively. These values are used to compute the “softmax” assignment of ***c***, such that cluster(***c***) = *arg max*_*i*_ *p*_*i*_ if *arg max*_*i*_ *p*_*i*_* *> 1.2*(1/r). Otherwise ***c*** is classified as ‘Unassigned’.
3. After determining that the initial classifier *C*_*R*_^*0*^ faithfully learns the reference clusters, we trained a classifier *C*_*R*_ on 100% of the cells in *R*. *C*_*R*_ was then applied to each cell ***v* **∈ *T* to generate predicted labels cluster(***v***). In this study, *T* was P12, P22 10d AWD, P22 1d RWD, and V1 P21.

The cell type frequency distribution predicted for *T* was compared to the distribution of cell type labels in *R* using scatter plots. Each dot represented one of the clusters in *R*, and the axes represented the frequency of that cluster in each dataset.

#### Principal component analysis on L2/3 pyramidal neurons.

The classification analyses revealed that L2/3 was the only subclass where cell type frequency differed significantly between P12 and P22 (Figs 3B and S3C). To explore this further, we performed PCA on L2/3 cells only. Since we were interested in understanding L2/3 cell type identity and how it varies across conditions, we chose as the features the top markers for each type (Wilcoxon rank-sum test, FC > 1.5, FDR < 0.05, expressed in >20% of cells in type), resulting in a set of 489 genes. The same set of genes was used for the PCA of all conditions. Correlations between the principal eigenvectors across the conditions were computed by taking their dot product. This is equivalent to the correlation coefficient of the loadings. Note that, by construction, the principal eigenvectors are orthonormal within a sample. Finally, a score for each marker set was computed for each cell as the mean expression of all genes in the set in that cell.

#### Overlap of tDE genes between S1 and V1.

To determine the degree to which tDE genes are shared between S1 and V1, we performed subclass-by-subclass differential gene expression on P14 and P21 V1 data, as they are the most closely matched to our P12 and P22 data [10]. We then performed a hypergeometric test for the overlap of tDE genes in either direction for each subclass. In each test for each subclass, four variables are set: *N,* the universal set comprising all of the genes downregulated/upregulated in every subclass, *K*, the number of tDE genes in V1, *n*, the number of tDE genes in wS1, and *k*, the intersections of *K* and *n.* The *P*-value for each subclass was Bonferroni-corrected by multiplying by the number of subclasses tested.

### Experimental methods

#### Mice handling.

All procedures were approved by the University of California, Berkeley Animal Care and Use Committee and were in accordance with National Institutes of Health (NIH) guidelines under Animal Use Protocol ID Number AUP-2016-02-8351-3. C57Bl6J male mice were obtained from Charles River Laboratories. Mice were maintained on a 12-h day/night cycle and housed with littermates and the mother in the UC Berkeley animal facility. For WD, mice were anesthetized with isoflurane, and whiskers were carefully plucked under a dissecting microscope with forceps using slow and steady force to prevent removal of the whisker follicle. Sham mice were anesthetized for the same amount of time as deprived, but whiskers were only stroked briefly with forceps.

#### Droplet-based snRNA-seq.

Mice were anesthetized with isofluorane, rapidly decapitated, and the brain was dissected out into ice-cold Hibernate A (BrainBits Cat# HACA). For each condition (P12, P22 control, P22 1d RWD, P22 10d AWD) 3 mice were used for each biological replicate of single-nucleus(sn) RNA-sequencing. Extracted brains were placed on a metal brain mold (Zivic Instruments,#5569) and the slice containing wS1 was isolated by inserting in the 11th space on the mold (~7.5 mm from the tip of the olfactory bulb, and a second blade 2 mm further anterior (4 spaces on the mold). This slice was removed and lowered to Hibernate A in a 60cc petri dish, placed on a ruler under a dissecting microscope. The midline was aligned with the ruler, and the first cut was bilaterally 2 mm out from the midline in a radial direction. The second cut was 2 mm medial to the first cut. The cortex was peeled off the underlying white matter. The wS1 piece was transferred into a RNAse-free cryovial, excess liquid was removed, and the tube was rapidly frozen on dry ice. Once all dissections were complete, the tissue was transferred from dry ice into a dewar of liquid nitrogen for storage before nuclei isolation.

#### Nuclei isolation.

Nuclei were isolated using the 10× Chromium nuclei isolation kit (10× Genomics, Cat#1000494). After isolation according to the chromium protocol, cells were counted on a hemocytometer in ethidium bromide and then diluted to 700–1,200 nuclei/mm^3^. Nuclei from each biological replicate were split into two tubes and run separately on two channels of 10× v3, targeting 10,000 cells per channel. We refer to these as library replicates. For each experiment, we performed two or three biological replicates towards a total of four to six library replicates.

#### Fluorescence in situ hybridization (FISH).

C57BL/6 male mice (Charles River), from age P12 and P22, were anesthetized with isoflurane and transcardially perfused with 2% RNAse-free paraformaldehyde (PFA) in PBS (pH 7.4). Mouse brains were collected and immediately fixed in 4% RNAse-free PFA at 4 °C. After 24 h, the brains were transferred to 30% sucrose in PBS and were allowed to sink 4 °C. Slices were cut from the left hemisphere in the ‘across-row’ plane. Brains were first mounted on a tissue guillotine with a 35 ° incline, rostral pointing upward. Brains were then cut at an angle 50° from the midsagittal plane. Using this plane, every S1 slice has one column from each whisker row A–E, and circuitry within columns remains largely intact [39,73]. Slices were cut on a sliding microtome (Reichert Scientific Instruments 860) into 30 µm thick sections, collected into RNAse-free PBS, and onto charged microscope slides. Sections were air-dried overnight, then post-fixed in 4% RNAse-free PFA for 1 h at 4°C; this was followed by serial ethanol dehydration and dried before being promptly stored at −80°C until further processing.

Multiplex FISH followed the protocol for ACDBio’s RNAscope Multiplex Fluorescent V2 Assay (Advanced Cell Diagnostics, cat# 323110). Thawed sections underwent H_2_O_2_ permeabilization, 5-min target retrieval, and protease III treatment. RNAscope probes *Trpc6* (cat# 442951), *Chrm2* (cat# 495311-C2), *Cdh13* (cat# 443251-C3), *Slc17a7* (cat# 501101-C4), *Adamts2* (Cat# 806371-C3)*, Bdnf* (Cat# 424821) *Btg2* (cat# 483001), *Npas4* (cat# 423431-C2), *Junb* (cat# 584761-C3), *Col19a1* (cat# 539701), *Sorcs3* (cat# 473421-C2), *Etv1* (cat# 557891-C3), and *Gabrg3* (cat# 514421-C4), were applied and amplified in sequence with TSA Vivid and Opal Polaris dyes (Advanced Cell Diagnostics, cat# 323271, 323272, 323273; Akoya Biosciences, cat# FP1501001KT). Cellular nuclei were counterstained with 1 µg/ml DAPI and mounted with Prolong Glass Antifade Mountant (Thermo Fisher Scientific, cat# P36982). All RNAscope runs were performed with both conditions side-by-side and controls, to reduce variability.

Imaging was performed using a Zeiss Axio Scan 7 slide scanner with Zen digital imaging software. Tilescan images were acquired of the entire wS1 including all cortical layers at 20×. All channels were acquired at the same exposure, gain, and laser power settings for each condition. The relevant barrel columns were identified using DAPI staining in combination with the other markers, and images were cropped to target L2/3 and barrel columns of interest in FIJI. All pre-processing steps were kept consistent between conditions. Cropped images were inputted into CellProfiler to detect 5 imaging channels. Nuclear segmentation was performed with DAPI channel, and cellular segmentation was performed with the RNAscope probe *Slc17a7* (vGlut1), a marker for glutamatergic neurons. For analysis of temporally-regulated mRNAs, nuclear segmentation with DAPI was used as a region of interest (ROI) to measure mean intensity values for each mRNA in each nucleus. Layers were estimated using empirically measured distance from the pial surface. L2/3 was considered 50–250 µm deep, and L4 was 250–350 µm. For cell type analysis, thresholds were set to identify cells expressing markers above background. Threshold parameters for each channel were kept the same across conditions. To determine cell type identity and overlap, cell type markers were identified as objects. Objects that did not overlap with the cell segmentation marker *Slc17a7* were eliminated in order to isolate excitatory L2/3 cells. Lastly, object overlap across channels was computed. The coordinates of all identified objects from CellProfiler were used to generate scatterplots in Python with the Seaborn package. For IEG analysis, cellular segmentation with *Slc17a7* was used as a ROI to measure intensity values for each mRNA in each excitatory L2/3 cell. Outliers were cleaned from the data in Graphpad Prism using the ROUT method [74]. Outline plots were generated in CellProfiler [75], by relating cellular segmentation object results and IEG objects. Outlines for each image used in the analysis were overlaid by z-projecting in FIJI to generate summary outline figures. Plots and statistics were generated using Graphpad Prism 10. Normality tests were performed on all datasets. If datum were not normally distributed, nonparametric statistical tests were used. Each condition (P12, P22 control, P22 1d sham, P22 1d RWD) had 3–5 mice from which multiple brain sections were analyzed.

## Supporting information

S1 Fig Data filtering steps, quality control, and gene ontology (GO) analysis of temporally regulated genes. **(A)** Bar plots showing the number of nuclei remaining in each biological replicate at the end of each filtering step (see Materials and methods for details). Biological replicates (x-axis) are colored by their experimental condition (legend, right). “PreQC” represents the default number of nuclei the 10× CellRanger software provides. “QC4” represents the final set of nuclei used for downstream analyses (S2 Data). **(B)** Distribution of total RNA counts detected in each biological replicate from each condition (S2 Data). **(C)** Distribution of total number of genes detected in each biological replicate from each condition (S2 Data). **(D)** The top 20 “biological process” gene ontology terms for Q1-Q4 for glutamatergic subclasses as shown in Fig 2D (S2 Data).
(PDF)

S2 Fig Developmental gene expression changes are subclass-specific. **(A)** UpSet plot [72] showing the overlap of GO terms associated with “biological process” (BP) across Q1-Q4 for glutamatergic neuronal subclasses. The lower panel indicates the set intersections corresponding to each column (e.g., the third column indicates the number of GO terms found in Q3 and Q4, but not in Q1 and Q2) (S2 Data). **(B)** Top GO terms enriched in Q1 (*left*) and Q3 (*right*) for GABAergic neuronal subclasses (S2 Data). **(C)** UpSet plots showing that downregulated (*left*) and upregulated (*right*) tDE genes between P12 and P22 are primarily subclass-specific. Only set intersections containing at least four genes are shown. Note that, unlike panel A, the sets here correspond to groups of subclasses rather than groups of quadrants (S2 Data). **(D)** Bar plots summarizing that ~60% of genes are regulated in only one subclass and that the number of downregulated genes is ~ 1.6× that of upregulated genes (S2 Data). **(E)** Visualization of the subclass-specific and global P12 > P22 genes from panel A in the quadrant analysis of Fig 2B for glutamatergic neurons. **(F)** Same as panel E for P22 > P12 genes (S2 Data). **(G)** Representative widefield images of RNAscope positive control showing expected labeling pattern (S2 Data). **(H)** Representative widefield images of RNAscope negative control using nontargeting probes showing no signal as expected (S2 Data).
(PDF)

S3 Fig Neuronal cell types at P22 and developmental changes. **(A)** UMAP visualization of P22 wS1 cell types in glutamatergic (left) and GABAergic (right) neurons. **(B)** Dotplots showing top cell type markers within each subclass at P22. Within each dotplot panel, rows indicate cell types and columns indicate genes. The size of each circle corresponds to the % of cells with nonzero expression, and the color indicates average expression level (S2 Data). **(C)** Within GABAergic neurons (~20% of all neurons), all cell types have approximately the same relative frequency between P12 (y-axis) and P22 (x-axis). Pearson correlation coefficient between the relative frequencies is indicated on top (S2 Data). **(D)** Percent variance captured (y-axis) by each principal component (PC) within L2/3 neurons at P12 and P22 (colors). Note that PCA is performed independently on each dataset. For both ages, a spectral gap is observed after PC1 and PC2 (S2 Data). **(E)** Pair-wise Pearson *R* values between the first four principal eigenvectors between P12 and P22. The first two principal eigenvectors corresponding to PC1 and PC2, which dominate the variance, map 1:1 between both ages with a high correlation.
(PDF)

S4 Fig Developmental gene expression changes at cell type resolution. **(A)** Expression patterns of aggregate expression scores (y-axis) for_L2/3_A, L2/3_B, and L2/3_C cells along PC1 (x-axis). Curves correspond to P12 and P22. *P*-values are from a Kolmogorov–Smirnov test between the two ages (S2 Data). **(B)** Barplot showing that L2/3 has more markers that are tDE between P12 and P22 than the other subclasses (S2 Data). **(C)** Expression patterns of some L2/3 cell type-enriched genes along PC1 from Fig 3C. Genes are colored based on their type enrichment: A, green; B, orange; C, purple. Other genes are shown in S6 Fig (see below) (S2 Data). **(D)** Same as Fig 3C, with cells colored by expression levels of *Cdh13* (left), *Trpc6* (middle), and *Chrm2* (right), which are targeted for FISH experiments in Fig 3D–3F (S2 Data).
(PDF)

S5 Fig Representative FISH images of L2/3 cell type markers. **(A)** Representative images of *Cdh13* labeling at P12 (top row) and P22 (bottom row). Overlay with *Slc17a7* (vGlut1) shows that the majority of *Cdh13*-expressing cells in the middle of L2/3 do not colocalize with *Slc17a7* (white arrows) whereas the *Cdh13 +* cells along the Layer 1/2 border do coexpress *Slc17a7* (white arrowheads). (right) Inset from area inside white squares. **(B)** Widefield images of ‘across-row’ section (see Materials and methods for details) with wS1 and surrounding cortical areas at P12. Arrows indicate cortical regions outside of wS1 where labeling becomes denser. **(C)** Widefield images of ‘across-row’ section with wS1 and surrounding cortical areas at P22. Arrows indicate cortical regions outside of S1 where labeling becomes denser. **(D)** Representative FISH images of wS1 L2/3 labeling cell type markers *Adamts2, Bdnf,* and *Chrm2* at P12 and P22. **(E)** Quantification of the fraction of excitatory (*Slc17a7+*) L2/3 cells expressing one or more of markers *Adamts2*, *Bdnf*, and *Chrm2* at P12 and P22. *N* = 3–4 slices from 2 mice per time point (S2 Data).
(PDF)

S6 Fig Expression patterns at P12 and P22 along PC1 of L2/3 type-enriched genes related to transcription factors (TFs), cell adhesion molecules (CAMs), and ion channels (ICs). **(A)** Expression patterns of type-enriched TFs at P12 and P22 in L2/3 cells ordered by PC1 value (S2 Data). **(B)** Same as A for ICs (S2 Data). **(C)** Same as A for CAMs (S2 Data).
(PDF)

S7 Fig Temporal gene expression changes in V1, GO enrichment for shared and region-specific genes, and mapping analysis. **(A)** UpSet plot (as in S2C Fig) summarizing subclass-by-subclass tDE analysis of V1 data. Only combinations containing at least four genes are shown (S2 Data). **(B)** Barplots showing that as in the case of wS1 (S2D Fig), most downregulated (*left*) and upregulated (*right*) genes in V1 are subclass-specific (S2 Data). **(C)** Full list of GO terms enriched in shared downregulated (*left*) or upregulated (*right*) tDE genes between V1 and wS1 (S2 Data). **(D)** UMAP plots of V1 (rows 1 and 3) and wS1 (rows 2 and 4) data colored by V1 labels. V1 neuron labels are based on the published clustering in Cheng and colleagues [10], while wS1 neurons were labeled using a supervised mapping analysis (see Materials and methods).
(PDF)

S8 Fig Subclass-level gene expression changes between P22 10d AWD and P22 control. **(A)** GABAergic cell types have approximately the same relative frequency in P22 10d AWD (y-axis) and normal P22 (x-axis). Note that cell type frequencies are normalized within all GABAergic neurons (~20% of all neurons) (S2 Data). **(B)** Similar to S3D Fig, showing that PC1 and PC2 are sufficient to describe transcriptional variance within L2/3 in the normal P22 and P22 10d AWD datasets (S2 Data). **(C)** Heatmap of Pearson correlation between the principal eigenvectors (as in S3E Fig) showing that the first two principal eigenvectors map 1:1 between the two datasets. **(D)** L2/3 type A, B, and C marker scores plotted as a function of a cell’s position along PC1. *P*-values are based on a Kolmogorov–Smirnov test comparing the two conditions (S2 Data). **(E)** UpSet plots showing that the few genes upregulated by 10d AWD are predominantly subclass-specific (S2 Data). **(F)** Same as B but for genes downregulated by 10d AWD (S2 Data). **(G)** Bar plots highlighting the small number of genes regulated by 10d AWD. Scale for y-axis is the same as for S2D Fig for comparison (S2 Data).
(PDF)

S9 Fig 1d RWD has little effect on L2/3 cell type identity. **(A)** GABAergic cell types have approximately the same relative frequency at P22 1d RWD and P22 normal whisker experience. Representation as in S8A Fig (S2 Data). **(B)** Same as panel A, for glutamatergic cell types (S2 Data). **(C)** PC1 and PC2 are sufficient to describe transcriptional variance within L2/3 in the normal P22 and P22 10d AWD datasets (S2 Data). **(D)** Similar to S8C Fig, comparing principal eigenvectors between the P22 1d RWD and normal P22 datasets. The first two principal eigenvectors map 1:1. **(E)** Similar to Fig 5C comparing the PC1 versus PC2 distribution and type-specific scores between P22 1d RWD and normal P22 L2/3 datasets (S2 Data). **(F)** L2/3 markers genes, as in S8D Fig, are shown as a function of cells’ position along PC1 comparing patterns between normal P22 and P22 1d RWD (S2 Data). **(G)** L2/3 type A, B, and C marker scores plotted as a function of a cell’s position along PC1. *P*-values are from a Kolmogorov–Smirnov test between the two conditions (S2 Data).
(PDF)

S1 Data Source data for Figs 1–6.
(XLSX)

S2 Data Source data for S1–S9 Figs.
(XLSX)

S1 Table tDE and SV scores for every tested gene in glutamatergic and GABAergic neurons.
(XLSX)

S2 Table Subclass-by-subclass differential expression testing results between P12 and P22.
(XLSX)

S3 Table Cell type markers from each subclass at P22.
(XLSX)

S4 Table Subclass-by-subclass differential expression testing results between P14 and P21 V1 data.
(XLSX)

S5 Table Subclass-by-subclass differential expression testing results between P22 10d AWD and P22.
(XLSX)

S6 Table Subclass-by-subclass differential expression testing results between P22 1d RWD and P22.
(XLSX)

## Abbreviations

AWD: all-whisker deprivation
CAMs: cell adhesion molecules
DEGs: differentially expressed genes
FDR: false-discovery rate
FISH: fluorescence in situ hybridization
FC: fold-change
GEM: gene expression matrix
HVGs: highly variable genes
HKs: housekeeping genes
IEG: immediate-early genes
ICs: ion channels
OR: odds ratio
PCs: principal components
PCA: principal component analysis
ROI: region of interest
RWD: row-based deprivation
SV: subclass variability
tDE: temporal differential expression
TFs: transcription factors
WD: whisker deprivation
