# Supplementary figures and images for: Molecular states underlying neuronal cell type development and plasticity in the postnatal whisker cortex
Source: PLoS Biol. 2025 May 14;23(5):e3003176. doi: 10.1371/journal.pbio.3003176 (PMC12119026; doi:10.1371/journal.pbio.3003176)

**A**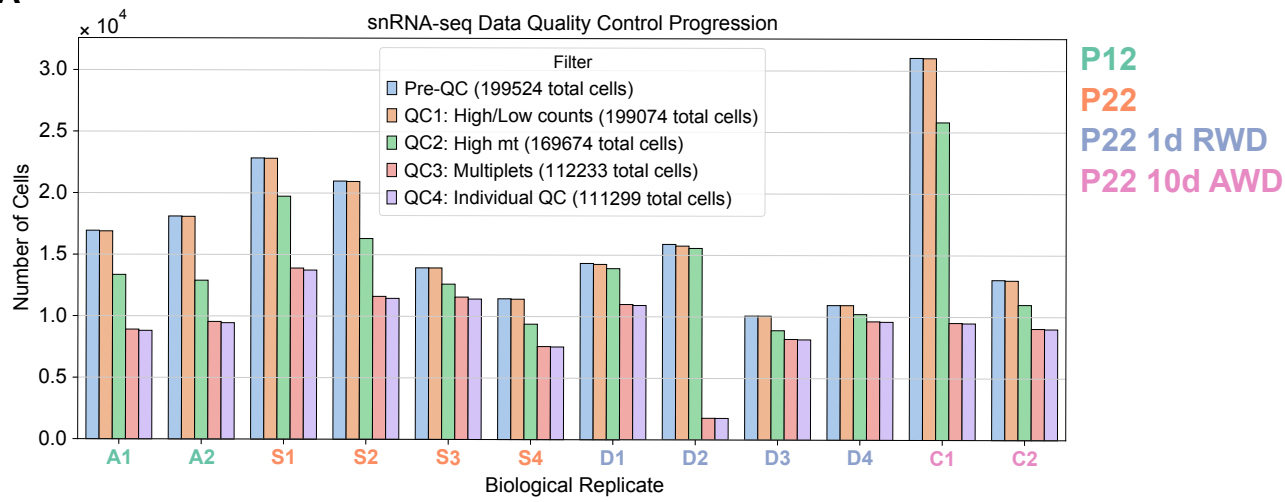**B**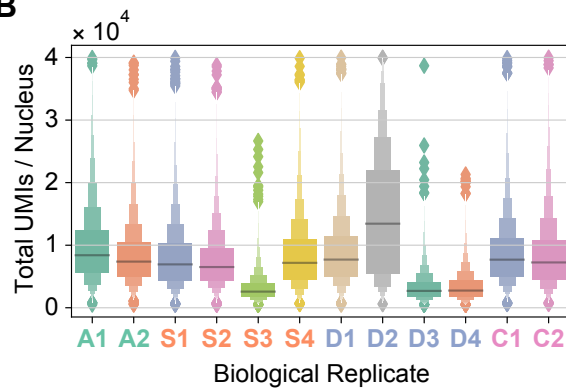**C**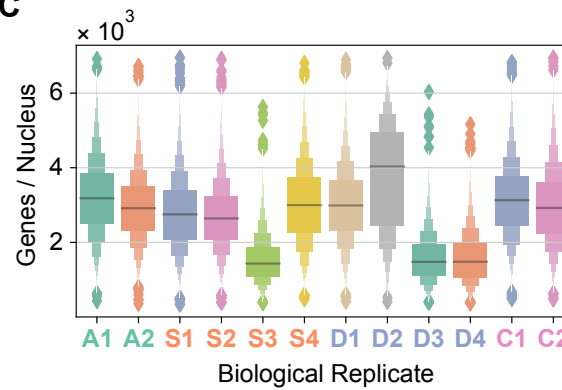**D**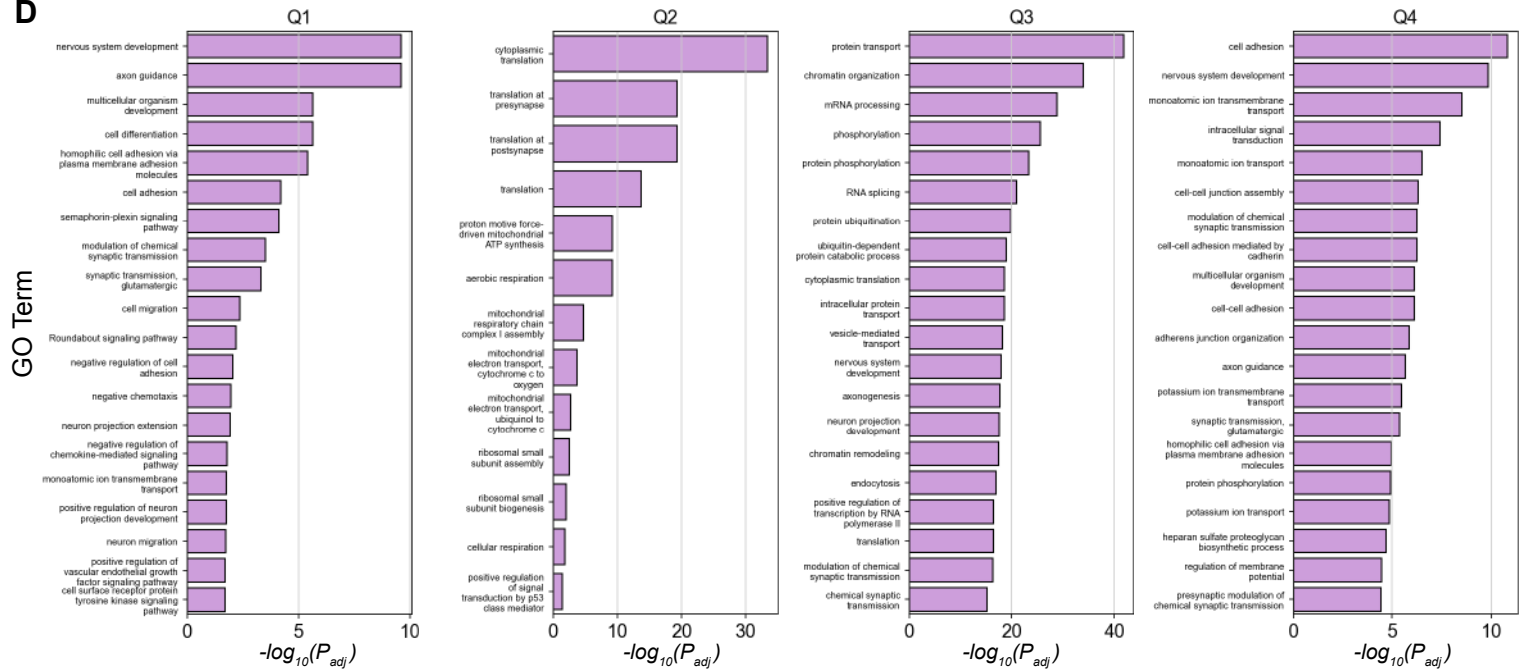

Supplement: S1 Fig — (A) Bar plots showing the number of nuclei remaining in each biological replicate at the end of each filtering step (see Materials and methods for details). Biological replicates (x-axis) are colored by their experimental condition (legend, right). “PreQC” represents the default number of nuclei the 10× CellRanger software provides. “QC4” represents the final set of nuclei used for downstream analyses (S2 Data). (B) Distribution of total RNA counts detected in each biological replicate from each condition (S2 Data). (C) Distribution of total number of genes detected in each biological replicate from each condition (S2 Data). (D) The top 20 “biological process” gene ontology terms for Q1-Q4 for glutamatergic subclasses as shown in Fig 2D (S2 Data). (PDF) [file pbio.3003176.s001.pdf]

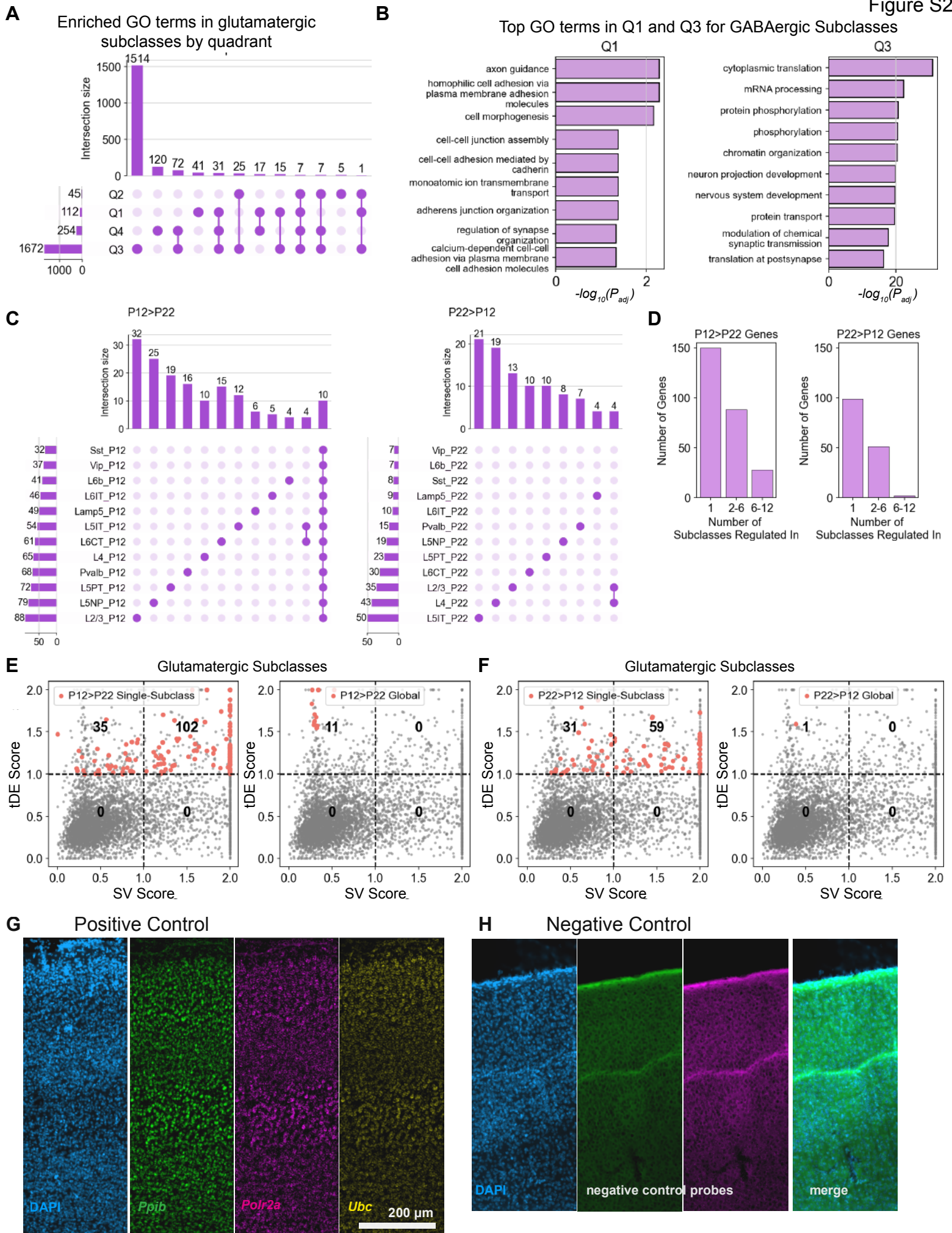

Supplement: S2 Fig — (A) UpSet plot [72] showing the overlap of GO terms associated with “biological process” (BP) across Q1-Q4 for glutamatergic neuronal subclasses. The lower panel indicates the set intersections corresponding to each column (e.g., the third column indicates the number of GO terms found in Q3 and Q4, but not in Q1 and Q2) (S2 Data). (B) Top GO terms enriched in Q1 (left) and Q3 (right) for GABAergic neuronal subclasses (S2 Data). (C) UpSet plots showing that downregulated (left) and upregulated (right) tDE genes between P12 and P22 are primarily subclass-specific. Only set intersections containing at least four genes are shown. Note that, unlike panel A, the sets here correspond to groups of subclasses rather than groups of quadrants (S2 Data). (D) Bar plots summarizing that ~60% of genes are regulated in only one subclass and that the number of downregulated genes is ~ 1.6× that of upregulated genes (S2 Data). (E) Visualization of the subclass-specific and global P12 > P22 genes from panel A in the quadrant analysis of Fig 2B for glutamatergic neurons. (F) Same as panel E for P22 > P12 genes (S2 Data). (G) Representative widefield images of RNAscope positive control showing expected labeling pattern (S2 Data). (H) Representative widefield images of RNAscope negative control using nontargeting probes showing no signal as expected (S2 Data). (PDF) [file pbio.3003176.s002.pdf]

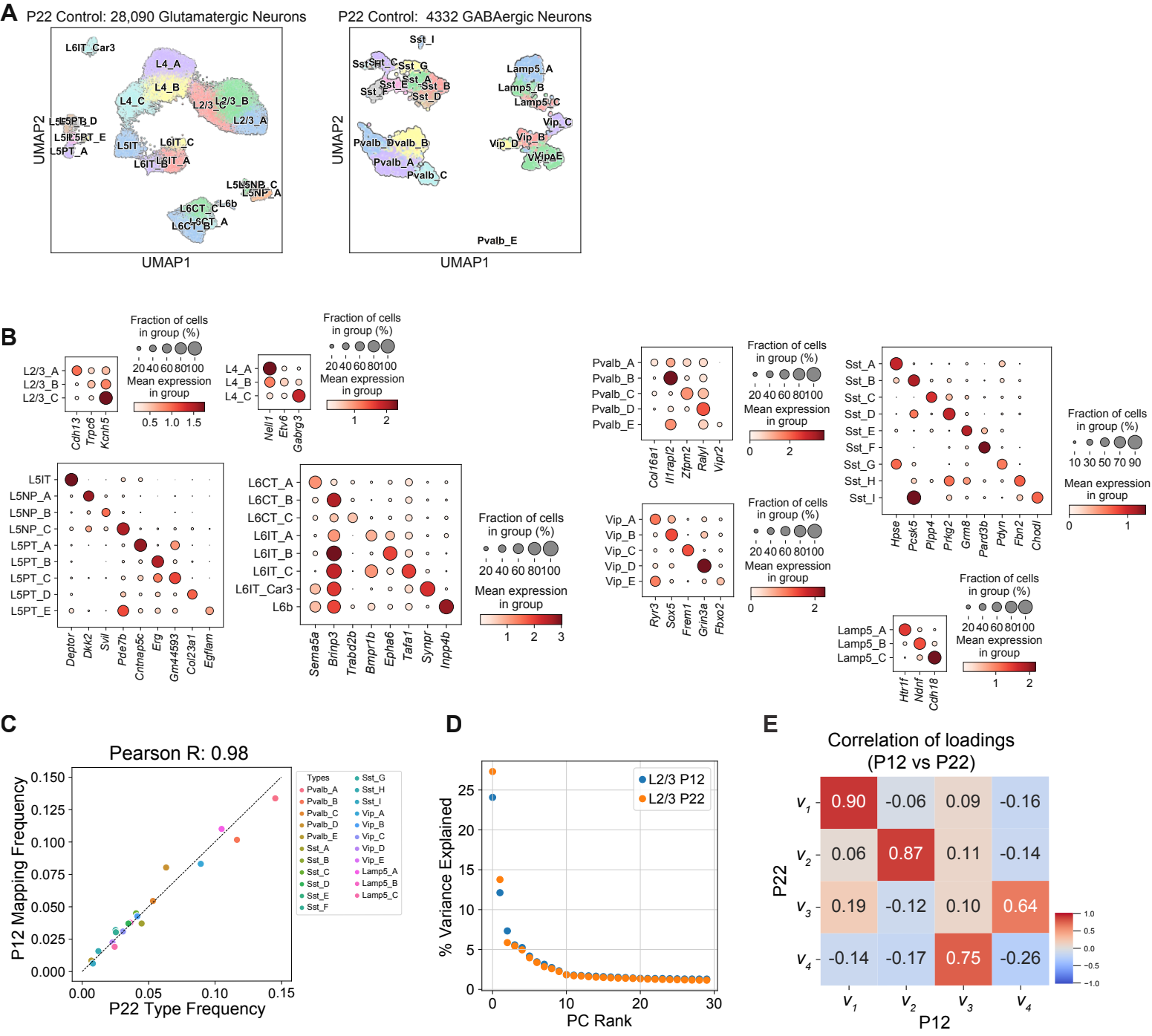

Supplement: S3 Fig — Neuronal cell types at P22 and developmental changes. (A) UMAP visualization of P22 wS1 cell types in glutamatergic (left) and GABAergic (right) neurons. (B) Dotplots showing top cell type markers within each subclass at P22. Within each dotplot panel, rows indicate cell types and columns indicate genes. The size of each circle corresponds to the % of cells with nonzero expression, and the color indicates average expression level (S2 Data). (C) Within GABAergic neurons (~20% of all neurons), all cell types have approximately the same relative frequency between P12 (y-axis) and P22 (x-axis). Pearson correlation coefficient between the relative frequencies is indicated on top (S2 Data). (D) Percent variance captured (y-axis) by each principal component (PC) within L2/3 neurons at P12 and P22 (colors). Note that PCA is performed independently on each dataset. For both ages, a spectral gap is observed after PC1 and PC2 (S2 Data). (E) Pair-wise Pearson R values between the first four principal eigenvectors between P12 and P22. The first two principal eigenvectors corresponding to PC1 and PC2, which dominate the variance, map 1:1 between both ages with a high correlation. (PDF) [file pbio.3003176.s003.pdf]

Figure S4

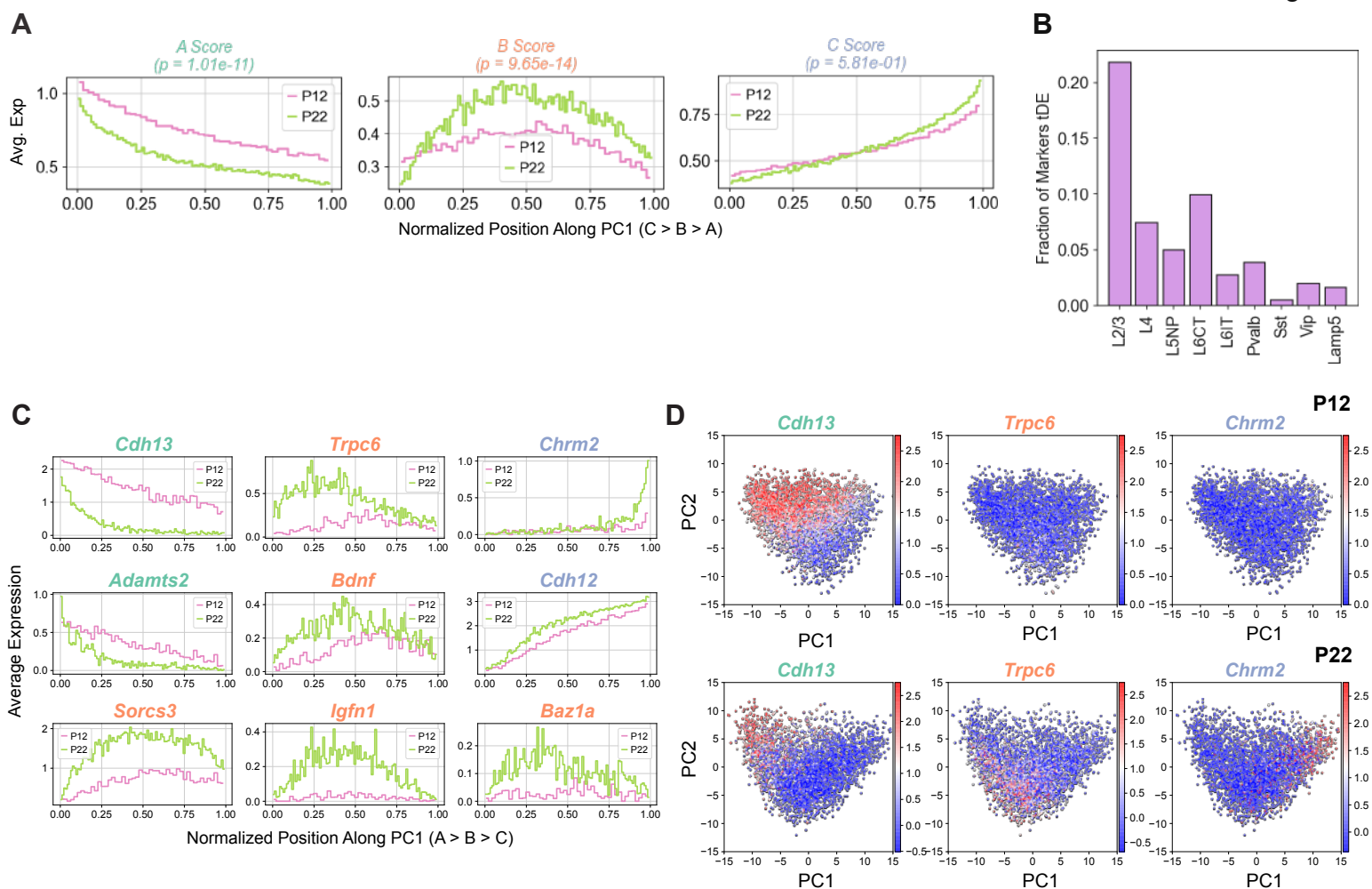

Supplement: S4 Fig — (A) Expression patterns of aggregate expression scores (y-axis) for_L2/3_A, L2/3_B, and L2/3_C cells along PC1 (x-axis). Curves correspond to P12 and P22. P-values are from a Kolmogorov–Smirnov test between the two ages (S2 Data). (B) Barplot showing that L2/3 has more markers that are tDE between P12 and P22 than the other subclasses (S2 Data). (C) Expression patterns of some L2/3 cell type-enriched genes along PC1 from Fig 3C. Genes are colored based on their type enrichment: A, green; B, orange; C, purple. Other genes are shown in S6 Fig (see below) (S2 Data). (D) Same as Fig 3C, with cells colored by expression levels of Cdh13 (left), Trpc6 (middle), and Chrm2 (right), which are targeted for FISH experiments in Fig 3D–3F (S2 Data). (PDF) [file pbio.3003176.s004.pdf]

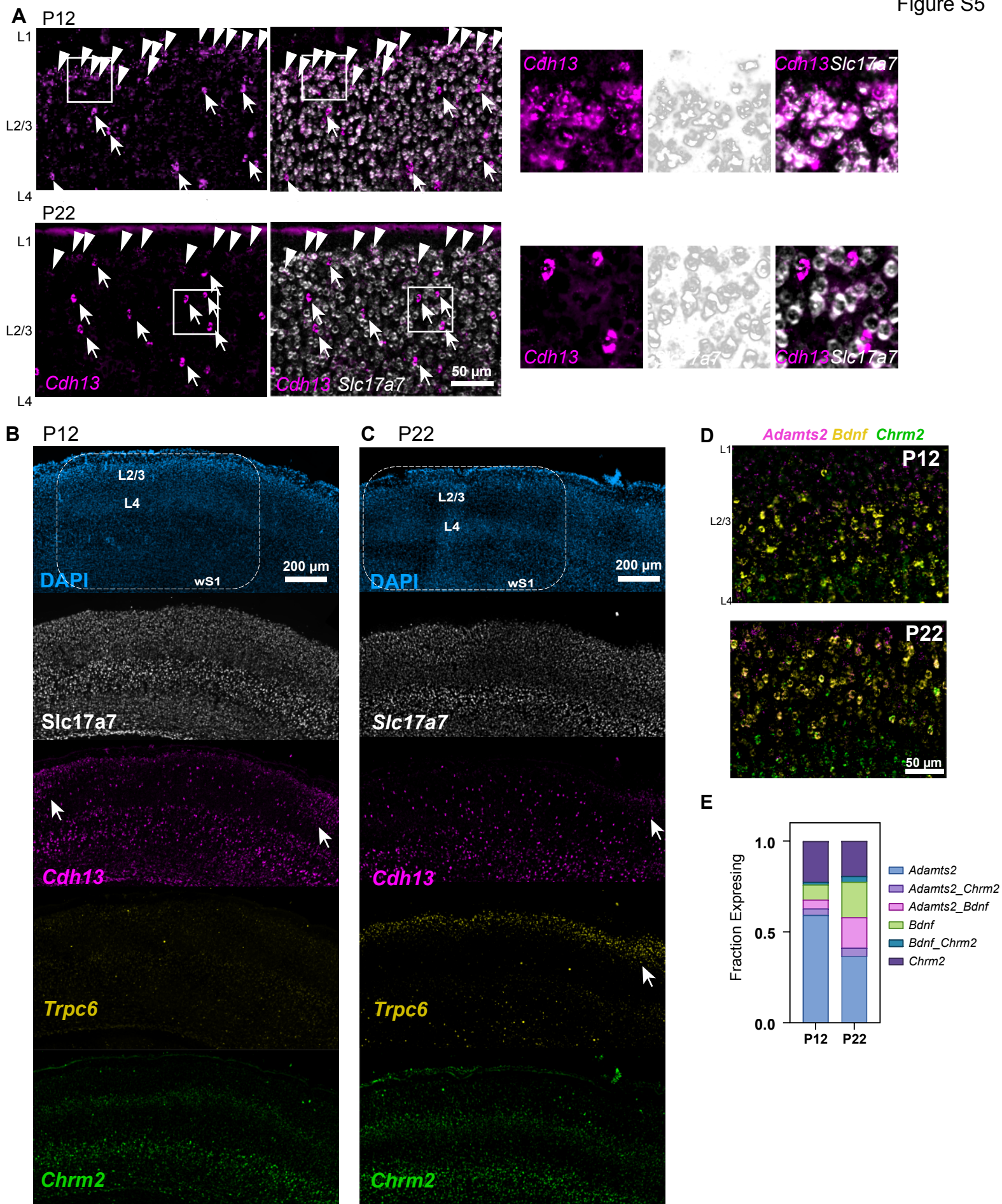

Supplement: S5 Fig — Representative FISH images of L2/3 cell type markers. (A) Representative images of Cdh13 labeling at P12 (top row) and P22 (bottom row). Overlay with Slc17a7 (vGlut1) shows that the majority of Cdh13-expressing cells in the middle of L2/3 do not colocalize with Slc17a7 (white arrows) whereas the Cdh13 + cells along the Layer 1/2 border do coexpress Slc17a7 (white arrowheads). (right) Inset from area inside white squares. (B) Widefield images of ‘across-row’ section (see Materials and methods for details) with wS1 and surrounding cortical areas at P12. Arrows indicate cortical regions outside of wS1 where labeling becomes denser. (C) Widefield images of ‘across-row’ section with wS1 and surrounding cortical areas at P22. Arrows indicate cortical regions outside of S1 where labeling becomes denser. (D) Representative FISH images of wS1 L2/3 labeling cell type markers Adamts2, Bdnf, and Chrm2 at P12 and P22. (E) Quantification of the fraction of excitatory (Slc17a7+) L2/3 cells expressing one or more of markers Adamts2, Bdnf, and Chrm2 at P12 and P22. N = 3–4 slices from 2 mice per time point (S2 Data). (PDF) [file pbio.3003176.s005.pdf]

Figure S7

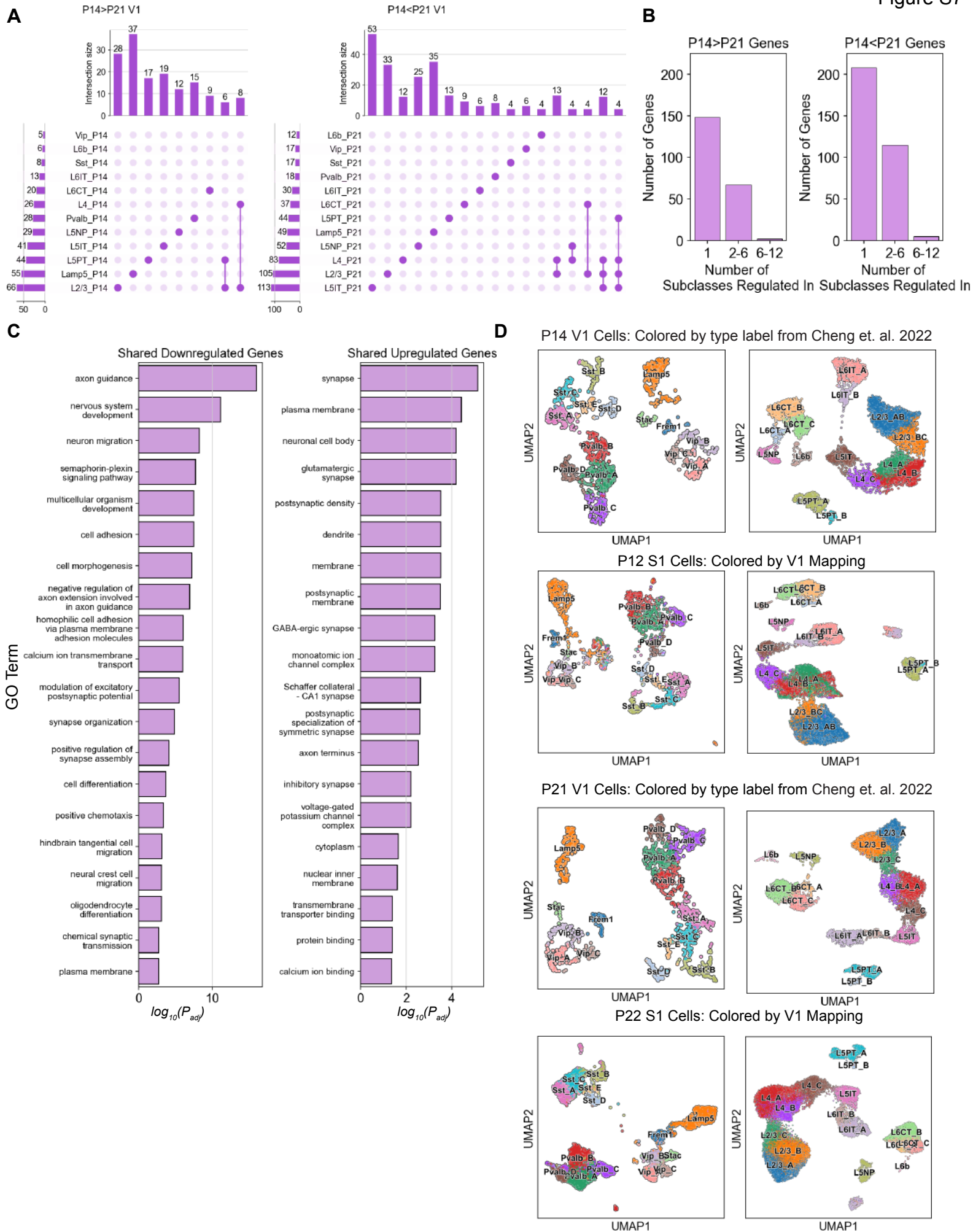

Supplement: S7 Fig — Temporal gene expression changes in V1, GO enrichment for shared and region-specific genes, and mapping analysis. (A) UpSet plot (as in S2C Fig) summarizing subclass-by-subclass tDE analysis of V1 data. Only combinations containing at least four genes are shown (S2 Data). (B) Barplots showing that as in the case of wS1 (S2D Fig), most downregulated (left) and upregulated (right) genes in V1 are subclass-specific (S2 Data). (C) Full list of GO terms enriched in shared downregulated (left) or upregulated (right) tDE genes between V1 and wS1 (S2 Data). (D) UMAP plots of V1 (rows 1 and 3) and wS1 (rows 2 and 4) data colored by V1 labels. V1 neuron labels are based on the published clustering in Cheng and colleagues [10], while wS1 neurons were labeled using a supervised mapping analysis (see Materials and methods). (PDF) [file pbio.3003176.s007.pdf]

Figure S8

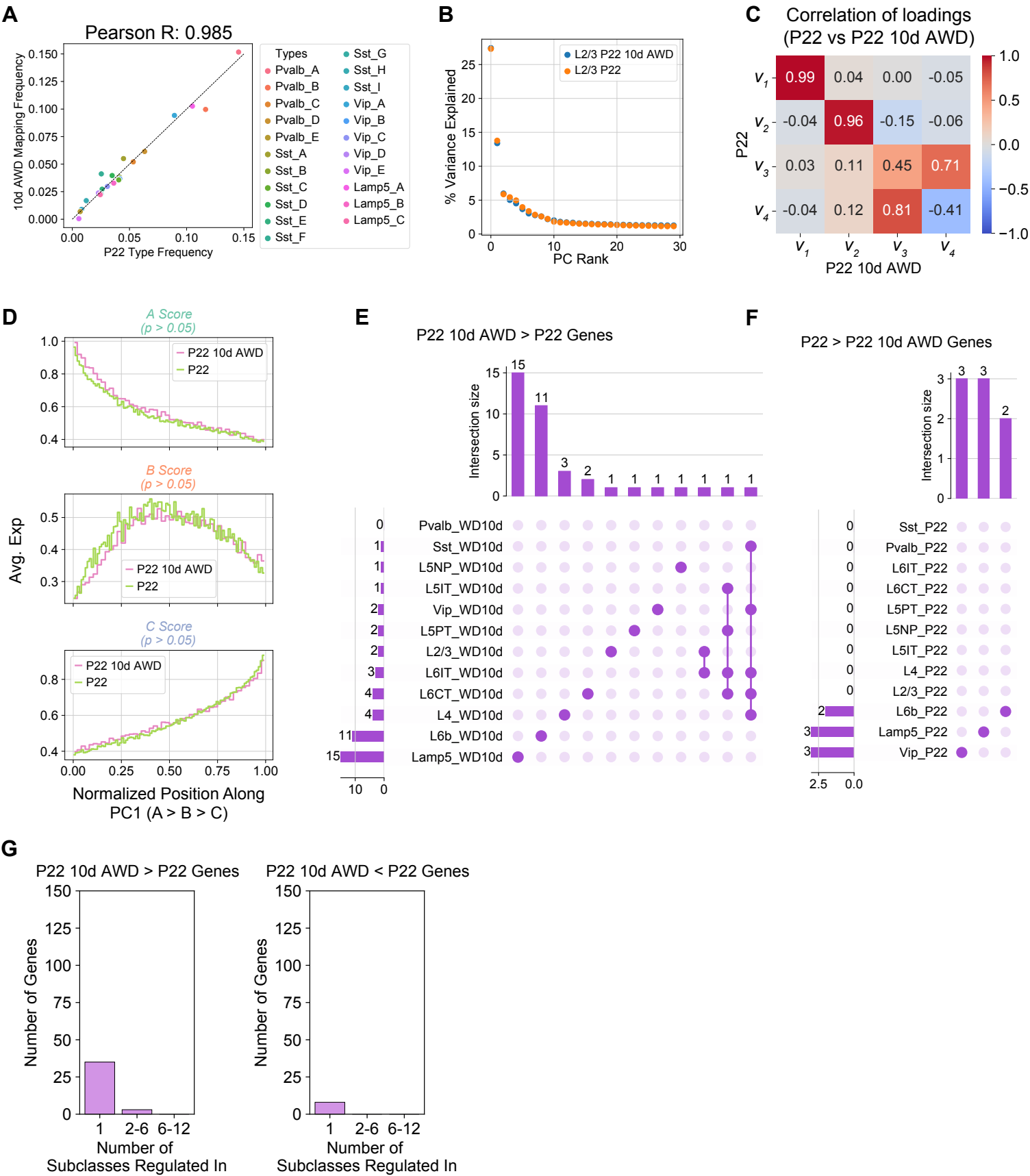

Supplement: S8 Fig — Subclass-level gene expression changes between P22 10d AWD and P22 control. (A) GABAergic cell types have approximately the same relative frequency in P22 10d AWD (y-axis) and normal P22 (x-axis). Note that cell type frequencies are normalized within all GABAergic neurons (~20% of all neurons) (S2 Data). (B) Similar to S3D Fig, showing that PC1 and PC2 are sufficient to describe transcriptional variance within L2/3 in the normal P22 and P22 10d AWD datasets (S2 Data). (C) Heatmap of Pearson correlation between the principal eigenvectors (as in S3E Fig) showing that the first two principal eigenvectors map 1:1 between the two datasets. (D) L2/3 type A, B, and C marker scores plotted as a function of a cell’s position along PC1. P-values are based on a Kolmogorov–Smirnov test comparing the two conditions (S2 Data). (E) UpSet plots showing that the few genes upregulated by 10d AWD are predominantly subclass-specific (S2 Data). (F) Same as B but for genes downregulated by 10d AWD (S2 Data). (G) Bar plots highlighting the small number of genes regulated by 10d AWD. Scale for y-axis is the same as for S2D Fig for comparison (S2 Data). (PDF) [file pbio.3003176.s008.pdf]

Figure S9

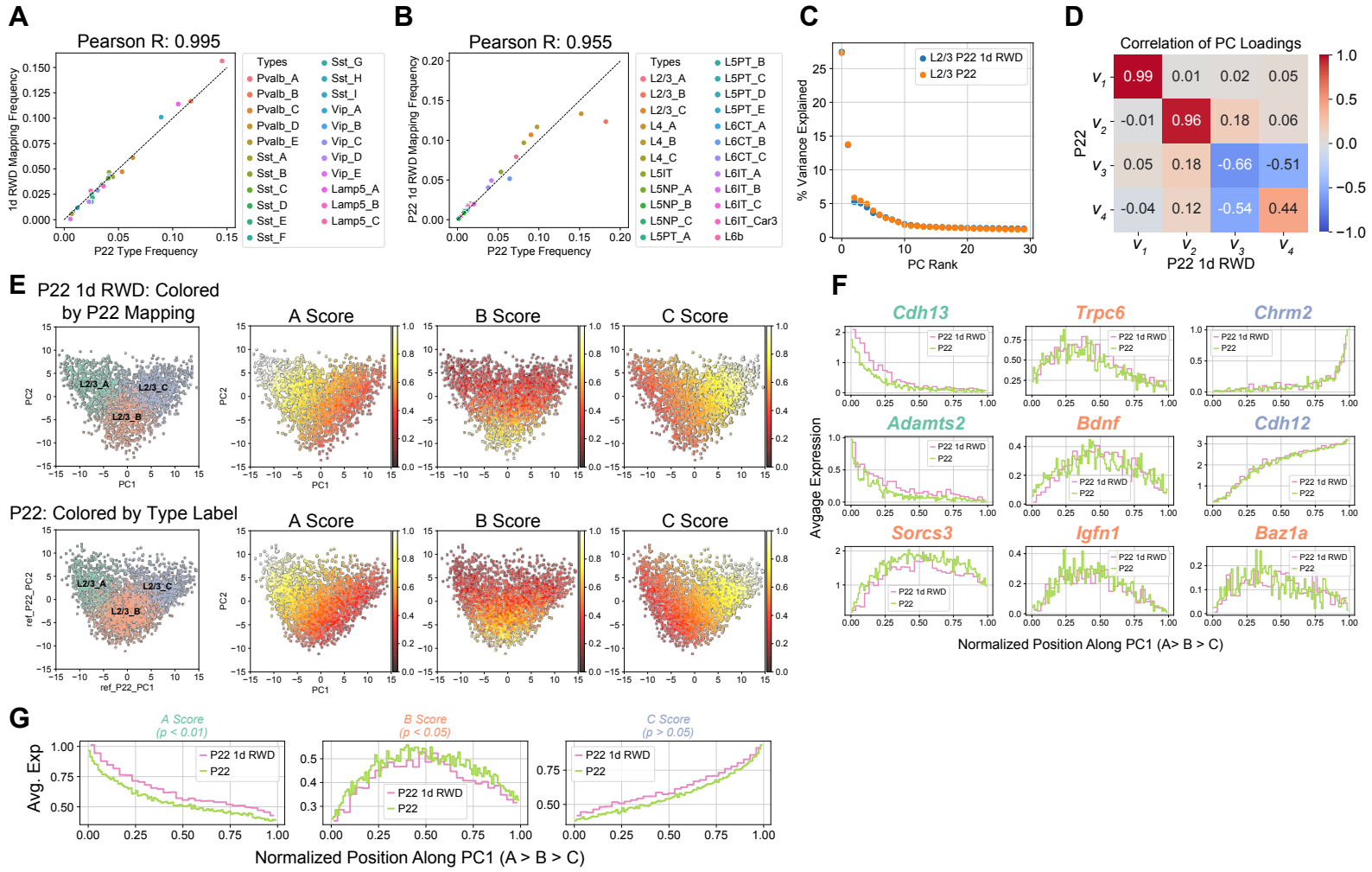

Supplement: S9 Fig — 1d RWD has little effect on L2/3 cell type identity. (A) GABAergic cell types have approximately the same relative frequency at P22 1d RWD and P22 normal whisker experience. Representation as in S8A Fig (S2 Data). (B) Same as panel A, for glutamatergic cell types (S2 Data). (C) PC1 and PC2 are sufficient to describe transcriptional variance within L2/3 in the normal P22 and P22 10d AWD datasets (S2 Data). (D) Similar to S8C Fig, comparing principal eigenvectors between the P22 1d RWD and normal P22 datasets. The first two principal eigenvectors map 1:1. (E) Similar to Fig 5C comparing the PC1 versus PC2 distribution and type-specific scores between P22 1d RWD and normal P22 L2/3 datasets (S2 Data). (F) L2/3 markers genes, as in S8D Fig, are shown as a function of cells’ position along PC1 comparing patterns between normal P22 and P22 1d RWD (S2 Data). (G) L2/3 type A, B, and C marker scores plotted as a function of a cell’s position along PC1. P-values are from a Kolmogorov–Smirnov test between the two conditions (S2 Data). (PDF) [file pbio.3003176.s009.pdf]
