# Supplementary material for: Molecular states underlying neuronal cell type development and plasticity in the postnatal whisker cortex
Source: PLoS Biol. 2025 May 14;23(5):e3003176. doi: 10.1371/journal.pbio.3003176 (PMC12119026; doi:10.1371/journal.pbio.3003176)

Figure S6

A Genes encoding Transcription Factors (TFs)

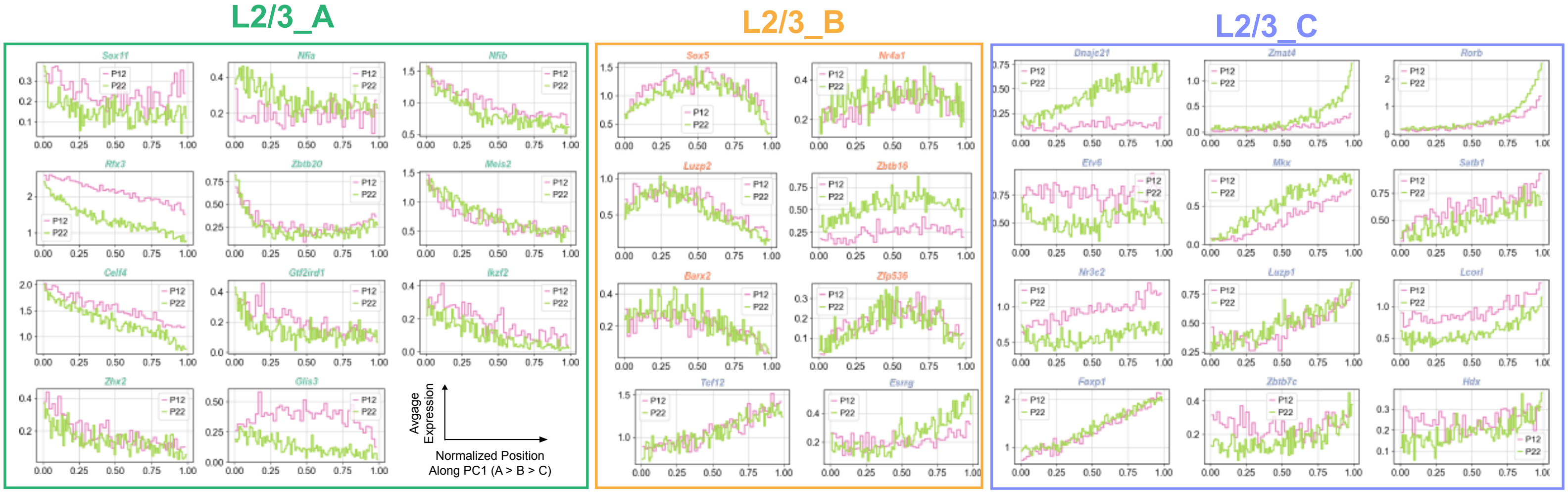

B Ion Channel related genes (ICs)

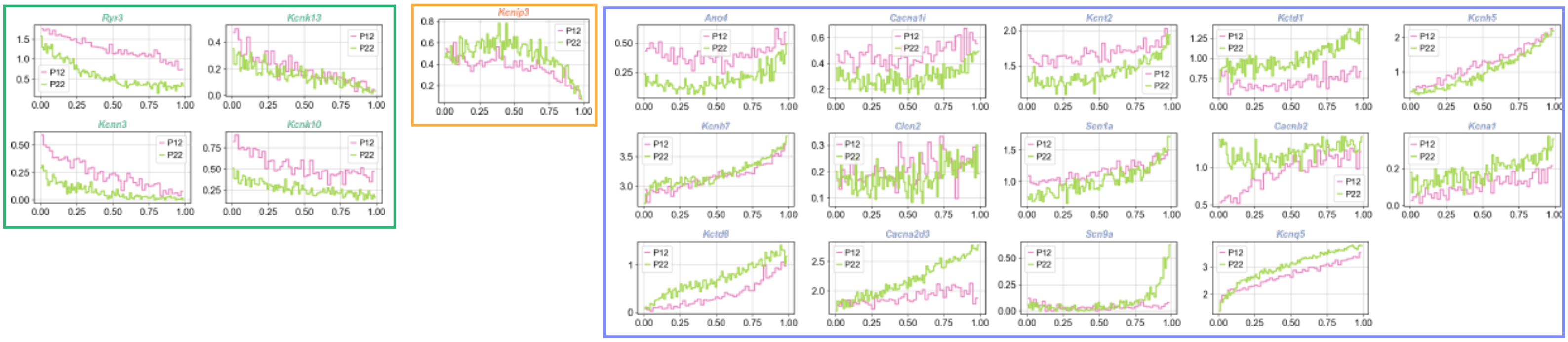

C Genes encoding cell adhesion molecules (CSMs)

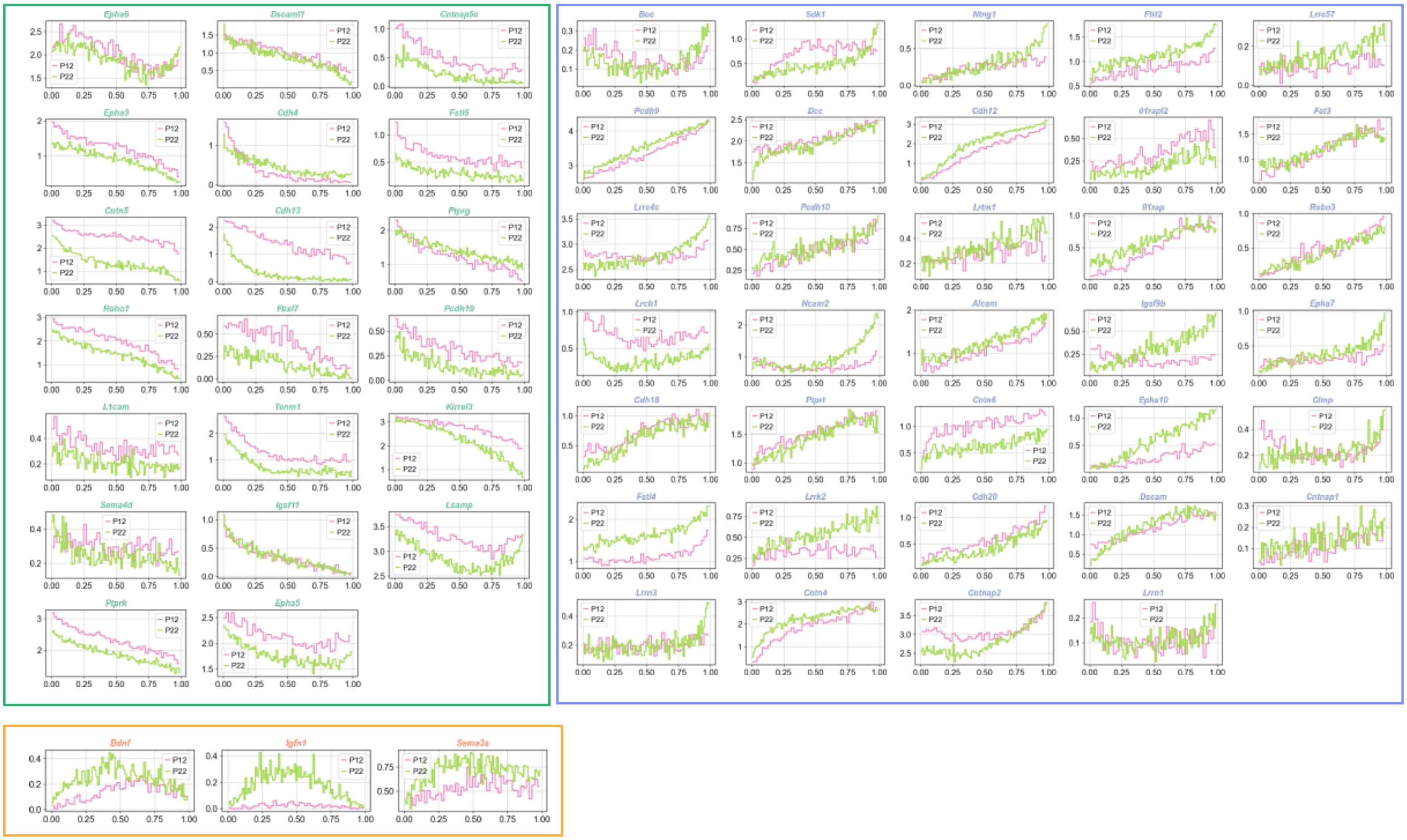

Supplement: S6 Fig — Expression patterns at P12 and P22 along PC1 of L2/3 type-enriched genes related to transcription factors (TFs), cell adhesion molecules (CAMs), and ion channels (ICs). (A) Expression patterns of type-enriched TFs at P12 and P22 in L2/3 cells ordered by PC1 value (S2 Data). (B) Same as A for ICs (S2 Data). (C) Same as A for CAMs (S2 Data). (PDF) [file pbio.3003176.s006.pdf]
